# Assessing the impact of arsenic metabolism efficiency on DNA methylation using Mendelian randomization

**DOI:** 10.1097/EE9.0000000000000083

**Published:** 2020-03-20

**Authors:** Anthony DiGiovanni, Kathryn Demanelis, Lin Tong, Maria Argos, Justin Shinkle, Farzana Jasmine, Mekala Sabarinathan, Muhammad Rakibuz-Zaman, Golam Sarwar, Md. Tariqul Islam, Hasan Shahriar, Tariqul Islam, Mahfuzar Rahman, Md. Yunus, Joseph Graziano, Mary V. Gamble, Habibul Ahsan, Brandon L. Pierce

**Affiliations:** a Department of Public Health Sciences, The University of Chicago, Chicago, Illinois; b Division of Epidemiology and Biostatistics, University of Illinois at Chicago, Chicago, Illinois; c UChicago Research Bangladesh, Mohakhali, Dhaka, Bangladesh; d Research and Evaluation Division, BRAC, Dhaka, Bangladesh; e International Centre for Diarrhoeal Disease Research, Bangladesh, Dhaka, Bangladesh; f Department of Environmental Health Sciences, Mailman School of Public Health, Columbia University, New York; g Department of Human Genetics; h Comprehensive Cancer Center, The University of Chicago, Chicago, Illinois; i Department of Medicine, The University of Chicago, Chicago, Illinois.

**Keywords:** Arsenic, Bangladesh, Epigenomics, DNA methylation, Mendelian randomization

## Abstract

Arsenic exposure affects >100 million people globally and increases risk for chronic diseases. One possible toxicity mechanism is epigenetic modification. Previous epigenome-wide association studies (EWAS) have identified associations between arsenic exposure and CpG-specific DNA methylation. To provide additional evidence that observed associations represent causal relationships, we examine the association between genetic determinants of arsenic metabolism efficiency (percent dimethylarsinic acid, DMA%, in urine) and DNA methylation among individuals from the Health Effects of Arsenic Longitudinal Study (n = 379) and Bangladesh Vitamin E and Selenium Trial (n = 393).

**Methods:**

We used multivariate linear models to assess the association of methylation at 221 arsenic-associated CpGs with DMA% and measures of genetically predicted DMA% derived from three SNPs (rs9527, rs11191527, and rs61735836). We also conducted two-sample Mendelian randomization analyses to estimate the association between arsenic metabolism efficiency and CpG methylation.

**Results:**

Among the associations between DMA% and methylation at each of 221 CpGs, 64% were directionally consistent with associations observed between arsenic exposure and the 221 CpGs from a prior EWAS. Similarly, among the associations between genetically predicted DMA% and each CpG, 62% were directionally consistent with the prior EWAS results. Two-sample Mendelian randomization analyses produced similar conclusions.

**Conclusion:**

Our findings support the hypothesis that arsenic exposure effects DNA methylation at specific CpGs in whole blood. Our novel approach for assessing the impact of arsenic exposure on DNA methylation requires larger samples in order to draw more robust conclusions for specific CpG sites.

## Abstract 2

Supplemental Digital Content is available in the text.

**What this study adds**

This article provides preliminary evidence supporting the hypothesis that many previously reported associations of arsenic exposure with DNA methylation at specific CpG sites represent causal relationships. Using a Mendelian randomization approach, we demonstrate that the directions of these previously reported associations are generally consistent with associations we estimate between genetically predicted arsenic metabolism efficiency and these arsenic-associated CpGs. Our work is unique because we apply Mendelian randomization to provide evidence supporting the hypothesis that findings from an epigenome-wide association study of an environmental exposure represent causal relationships, using genetic instruments that have well-established associations with arsenic metabolism efficiency.

## Introduction

Exposure to arsenic from consumption of naturally contaminated drinking water impacts >100 million people globally, including ~50 million in Bangladesh.^1^ Arsenic is a toxic and carcinogenic metal, and consumption of drinking water with arsenic concentrations above 50–100 µg/L is associated with risk for several cancer types in multiple populations.^2,3^ Arsenic exposure is also associated with increased risk of cardiovascular diseases,^4,5^ respiratory disease,^6^ diabetes,^7^ nonmalignant lung disease,^8^ and increased overall mortality.^9^ Chronically exposed individuals maintain high risks of arsenic-associated diseases and mortality for several decades.^10^ Thus, an understanding of arsenic toxicity mechanisms is needed to better assess risk and develop effective prevention and treatment strategies for arsenic-associated diseases and health effects.

The mechanisms of arsenic toxicity are complex and remain to be fully elucidated. Prior studies suggest that arsenic alters DNA methylation, and arsenic-associated DNA methylation alterations may be an important mechanism of arsenic toxicity.^11^ DNA methylation is an epigenetic modification that primarily occurs at cytosine nucleotides within CpG sites (CpGs) and reflects chromatin conformation and regulation of gene expression. Prenatal arsenic exposure is associated with CpG methylation in cord blood according to numerous studies,^12–16^ and differential methylation in blood has been found in adult Bangladeshi, American, and Argentinian populations with a wide range of exposure levels (measured in urine, from ~ 10 μg/g creatinine to >1600 μg/g creatinine).^17–20^ A recent epigenome-wide association study (EWAS) of Bangladeshi adults identified associations between urinary arsenic and methylation at >200 CpGs in whole blood.^21^ These CpGs are enriched in genes belonging to cancer, aging, inflammation, and toxicant response pathways.^21^ While a mechanism of arsenic-induced carcinogenesis involving DNA methylation changes is plausible, we do not know that associations observed in EWAS represent causal effects of arsenic on the epigenome.

Arsenic undergoes biotransformation by arsenite methyltransferase (*AS3MT*), and arsenic metabolism efficiency modulates internal dose of arsenic by facilitating excretion of arsenic in urine. *AS3MT* converts inorganic arsenic (iAs) via methylation reactions into monomethylarsonic acid (MMA) and then dimethylarsinic acid (DMA).^22,23^ The proportion of DMA among all arsenic species in urine (DMA%) is a measure of arsenic metabolism efficiency.^24^ These methylated species, especially DMA, are more readily excreted in urine than iAs, and higher DMA relative to MMA in urine is associated with lower arsenic toxicity risk.^25–28^ Therefore, arsenic metabolism efficiency reflects variability in the internal arsenic dose for individuals at a given level of exposure. Because DMA% has known genetic determinants, it is possible to use a Mendelian randomization (MR) approach to estimate associations between DMA% and methylation at specific CpGs that are not biased due to confounding by environmental or lifestyle factors. Genetic determinants of arsenic metabolism can be utilized as instrumental variables (IVs); and given that these IVs (1) affect arsenic metabolism and (2) are associated with CpG methylation exclusively through their effect on arsenic metabolism, MR provides accurate effect estimates.^29^ If these conditions are satisfied, MR estimates avoid biases that may affect associations with directly measured arsenic metabolism, such as confounding or reverse causation. Genome-wide association (GWA) and candidate gene studies have identified independent associations of variants in the 10q24.32/*AS3MT* region, rs9527 and rs11191527, with DMA% among arsenic-exposed Bangladeshi individuals.^24,30,31^ DMA% is also associated with rs61735836 in exon 3 of *FTCD* (Formiminotransferase cyclodeaminase), which has a catalytic role in the one-carbon/folate cycle, a source of methyl groups for arsenic methylation.^32^

**Figure 1.**
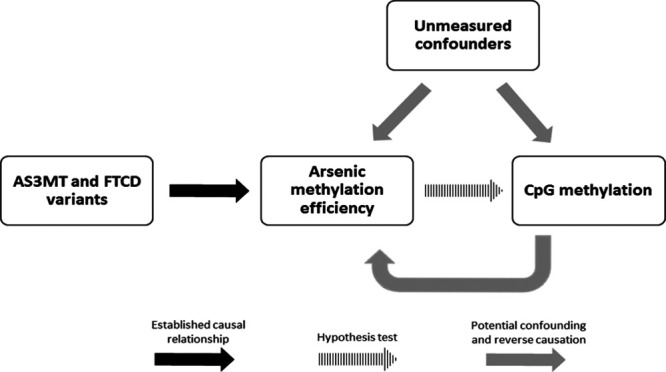
Causal diagram depicting the relationships among 10q24.32 and *FTCD* genetic variants, arsenic methylation efficiency, and methylation levels of CpG sites in an arsenic-exposed population. The causal relationship between the 10q24.32 and *FTCD* variants and arsenic methylation permits the use of these variants as potential IVs in our MR analysis.

In this study, we use an MR approach to evaluate the relationship between arsenic metabolism efficiency and methylation of arsenic-associated CpGs identified in prior EWAS.^21^ Efficient metabolism of arsenic (i.e., high DMA%) should reduce the internal dose of arsenic; thus, we hypothesize that DMA% will be associated with the CpGs discovered in EWAS in a direction opposite to that of arsenic exposure. Using data on 379 Bangladeshi participants in the Health Effects of Arsenic Longitudinal Study (HEALS) and 393 participants in the Bangladesh Vitamin E and Selenium Trial (BEST),^33,34^ we obtain MR-based estimates (Figure 1) of the association between arsenic metabolism efficiency and each arsenic-associated CpG, using genetic determinants of DMA% as IVs.^35^ Our approach represents a novel strategy to examine the toxicological relevance of molecular features showing association with environmental exposures, such as DNA methylation, in observational studies.

## Methods

### Study participants

Subjects analyzed in this article were participants in one of the two following studies: the HEALS and the BEST. DNA samples were obtained at baseline from participants in both cohorts. HEALS (described previously in detail) is a prospective longitudinal study of health outcomes associated with chronic arsenic exposure in Araihazar, Bangladesh, with 11,746 adults (11,224 with arsenic measurements) enrolled at the original baseline visit (age 18–75 years).^33^ Trained study physicians (blinded to arsenic measurements) conducted in-person interviews, clinical evaluations, and collection of urine and blood samples using structured protocols.^36^ BEST is a 2 × 2 factorial randomized chemoprevention trial assessing the effects of vitamin E and selenium dietary supplements on skin cancer risk among Bangladeshi individuals (n = 7,000, age 25–65 years) with arsenical skin lesions.^37^ Many of the study protocols in BEST, including sample collection and exposure assessment, were identical to those in HEALS.^24^ This article uses DNA methylation data from a recent EWAS of 396 randomly selected adults from HEALS, and from a prior study of 400 BEST participants.^19,21^ Study protocols were approved by the Institutional Review Boards of The University of Chicago and Columbia University, the Ethical Review Committee of the International Center for Diarrheal Disease Research, Bangladesh, and the Bangladesh Medical Research Council. Informed consent was obtained from all participants.

### Exposure assessment

Arsenic was measured in urine at baseline for HEALS and BEST participants and in drinking water for HEALS participants. At baseline, each HEALS participant identified the well used as their primary source of drinking water. Urinary and water arsenic were measured using graphite furnace atomic absorption spectrometry in a single laboratory.^38^ Total urinary arsenic concentration was divided by creatinine to compute creatinine-adjusted arsenic (micrograms/gram creatinine).^39^ In HEALS participants, urinary arsenic metabolites were distinguished via high-performance liquid chromatography, followed by detection using inductively coupled plasma-mass spectrometry with dynamic reaction cell.^26^ The percentages of iAs, MMA, and DMA among all arsenic species were calculated after subtracting arsenobetaine and arsenocholine (nontoxic organic arsenic from dietary sources) from total arsenic.

### Genotyping

DNA extraction and quality control for HEALS and BEST blood samples have been described in the respective studies in which the 10q24.32 and *FTCD* variant associations with arsenic metabolism efficiency were discovered.^24,32^ Genotypes for the 10q24.32 and *FTCD* variants were obtained from Illumina HumanCytoSNP-12 v2.1 chips and Illumina’s exome array v1.1, respectively. Genotypes for rs9527 and rs11191527 were available for 379 individuals in HEALS and 393 in BEST, and genotypes for rs61735836 were available for 340 individuals in HEALS and 383 in BEST (Table 1).

**Table 1.**
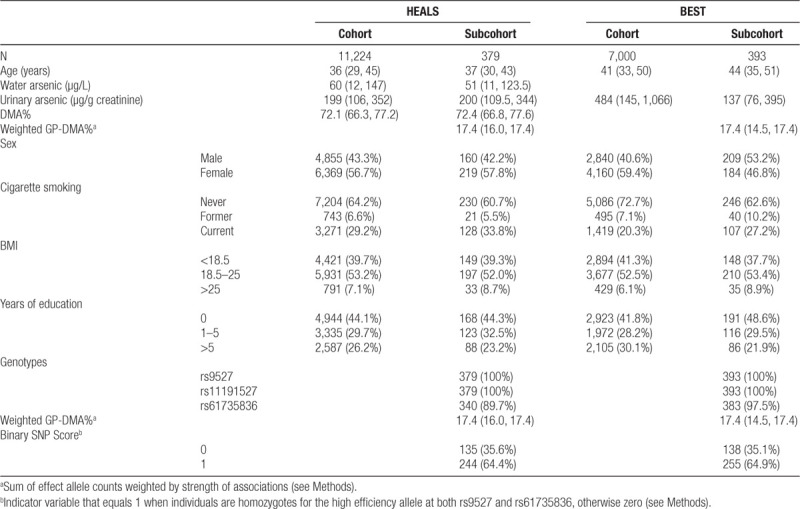
Descriptive statistics (median [25th,75th percentiles] or n [%]) for HEALS and BEST cohorts and subcohorts used for this analysis

### DNA methylation

DNA methylation was measured on the Illumina EPIC array for 396 HEALS participants and 450K array for 400 BEST participants.^21,37^ Preprocessing and normalization of these arrays has been described previously.^21^ Briefly, beta mixture quantile (BMIQ) normalization was applied to reduce type I/II probe bias, and an EWAS of log_2_-transformed urinary arsenic was conducted in both HEALS and BEST using models adjusted for age, sex, smoking status, BMI, and surrogate variables.^40^ The METAL software was used to conduct a meta-analysis using inputted summary statistics from 390,810 CpGs measured in both HEALS and BEST.^41^ Analyses of the EPIC array data from HEALS participants identified associations between log_2_-transformed urinary arsenic and 34 CpGs at FDR = 0.05 (eTable 1; http://links.lww.com/EE/A77). From the meta-analysis of both HEALS and BEST, we identified 221 CpGs associated with log_2_-transformed urinary arsenic at FDR = 0.05 (eTable 2; http://links.lww.com/EE/A77).

### Estimation of GP-DMA%

We generated a weighted SNP score representing genetically predicted DMA% (GP-DMA%) as a measure of arsenic metabolism efficiency. β coefficients for associations of rs9527, rs11191527, and rs61735836 with DMA% were obtained from prior GWA study in HEALS.^24,32^ The β coefficient for the association of each SNP with DMA% (Table 2) was multiplied by its high-efficiency allele (effect allele) count, and the score was computed as the sum of these products. GP-DMA% captured the increase in DMA% relative to an arbitrary baseline DMA%. Let X_j_ be the high-efficiency allele count of each *j*th SNP, and β_Xj_ be the effect size of the association between SNP *j* and DMA%. Then:

**Table 2.**
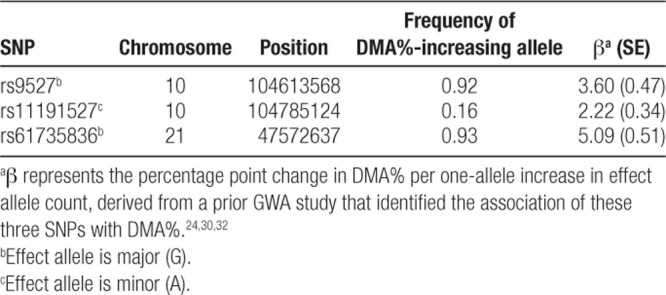
SNPs used as IVs for arsenic metabolism efficiency were selected based on their association with DMA% in prior GWA studies


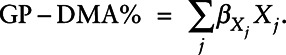


In a supplementary analysis, we defined a binary score indicating whether each individual carried only high-efficiency alleles at both rs9527 and rs61735836 (or if they carried at least one low-efficiency allele at one or both of these SNPs). This strategy avoided specifying weights, rather relying on the two SNPs with (1) large effect sizes (compared with rs11191527) and (2) low frequency of the low-efficiency allele (Table 2).

### Statistical methods

Associations of CpG methylation with DMA%, 10q24.32 and *FTCD* SNPs, and GP-DMA% were estimated by linear regression. Because of small sample sizes of minor allele homozygotes among our SNPs (at most 2.3% of the sample), these individuals were combined with heterozygotes and classified as minor allele carriers for analyses of single SNPs. Each SNP genotype was coded as a binary variable for higher effect allele count. Because of moderate LD between rs9527 and rs11191527, these SNPs were included together as covariates in individual SNP analyses. All regressions included age, sex, methylation batch, and cigarette smoking status (never, former, and current smoker) as covariates. Analyses in which HEALS and BEST were combined were adjusted for cohort (HEALS or BEST). Regressions with DMA% as a predictor (restricted to HEALS) also included BMI, years of education, and log-transformed water arsenic as covariates, to adjust for potential confounders of this non-genetic variable. As an additional adjustment for confounding due to arsenic exposure levels, associations between log-transformed DMA and CpG methylation were adjusted for log-transformed urinary arsenic and log-transformed urinary creatinine in addition to the same covariates as in DMA% regressions.

**Figure 2.**
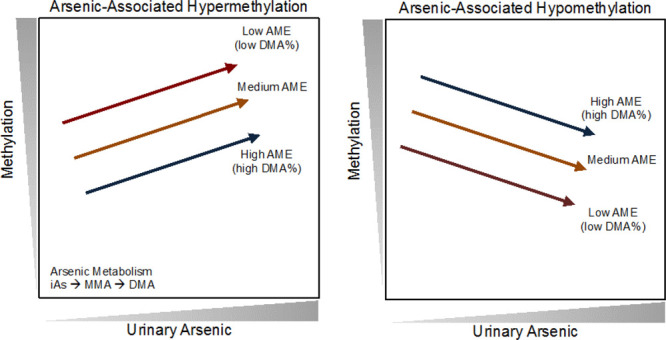
Hypothesized relationships among arsenic metabolism efficiency (AME), urinary arsenic, and CpG methylation. For CpGs that show a positive association with urinary arsenic (hypermethylated CpGs, left panel), higher AME should reduce the internal dose of arsenic and thus show a negative association with CpG methylation. For CpGs negatively associated with urinary arsenic (hypomethylated CpGs, right panel), higher AME should show a positive association with CpG methylation.

Due to low power for testing the association of SNPs (or DMA%) with any specific CpG site, we tested the hypothesis that the set of arsenic-associated CpGs tended to associate with DMA% (and with genotypes that impact DMA%) in directions consistent with the CpGs’ associations with arsenic. We applied binomial tests to binary variables representing the directional consistency of association for each CpG site. For each CpG, the estimate of association with DMA% or GP-DMA% (or the MR estimate) was considered consistent with prior EWAS if the β coefficient had a direction opposite to that of the previously reported association between arsenic exposure and CpG methylation. In other words, we hypothesized that if arsenic metabolism efficiency impacts DNA methylation, increased DMA% (or GP-DMA%) would lower the internal arsenic dose, thus the direction of association for each arsenic-associated CpG site would be opposite to that of arsenic exposure (Figure 2). Under the null hypothesis, the number of CpGs showing a direction of association consistent with the prior EWAS is equal (or less than) to the number of CpGs with a direction of association that is inconsistent with the prior EWAS. We report one-sided *P*-values (from a binomial test) that correspond to the alternative hypothesis the number of CpG showing an association directionally consistent with prior EWAS is greater than the number of CpGs showing a directionally inconsistent association.

### Mendelian randomization

MR estimates were computed using the “Mendelian randomization” R package,^42^ using rs9527, rs11191527, and rs61735836 as IVs in the inverse-variance weighted (IVW) MR method. Maximum likelihood MR was also conducted as a sensitivity analysis. Effect sizes (β_Xj_) and standard errors (se(β_Xj_)) for associations between each *j*th SNP and DMA% were derived from prior publications from HEALS (Table 2).^24,32^ For SNP ~ CpG associations, effect estimates (β_Yj_) and standard errors (se(β_Yj_)) were obtained from the regression analyses conducted in this study.

## Results

### Participant characteristics

Genotyping and methylation data were available for 379 HEALS participants and 393 BEST participants. The median age in HEALS was 37 (IQR: 30, 43) and 44 years in BEST (IQR: 35, 51), with 57.8% women in HEALS and 46.8% women in BEST (Table 1). In HEALS, 33.8% were current smokers and 5.5% former smokers; and in BEST, 27.2% were current smokers and 10.2% former. The median urinary arsenic (adjusted for creatinine) was 200 µg/g (IQR: 109.5, 344) in HEALS and 137 µg/g (IQR: 76, 395) in BEST. Water arsenic and DMA% data are not available for BEST participants.

### Associations between CpG methylation and arsenic metabolism

Median DMA% in the HEALS participants was 72.4% (IQR: 66.8%, 77.6%) (Table 1). The correlation between DMA% and log-transformed creatinine-adjusted urinary arsenic was −0.16 (n = 383, *P* = 1.3 × 10^−3^), and between log-transformed DMA and log-transformed creatinine-adjusted urinary arsenic was 0.52 (n = 383, *P* = 1.8 × 10^−27^) (eFigure 1; http://links.lww.com/EE/A77).

**Figure 3.**
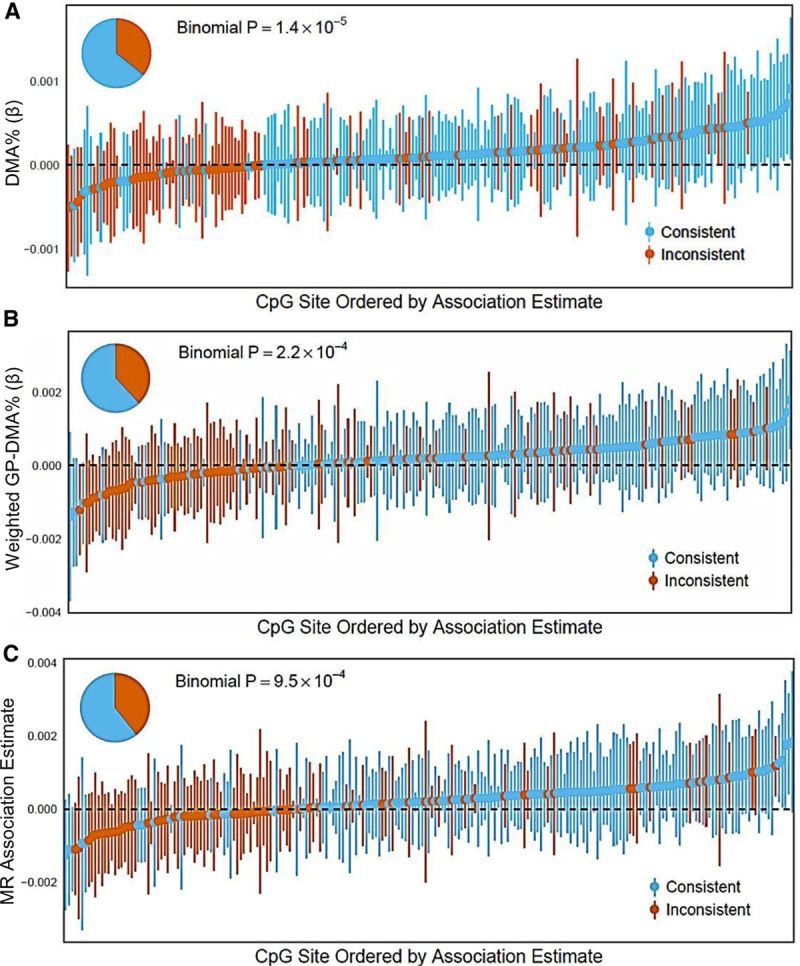
Associations of DMA% and genetically predicted DMA% with DNA methylation at 221 arsenic-associated CpGs discovered in meta-analysis. β coefficients for associations of (A) DMA% and (B) weighted GP-DMA% with CpG sites, as well as (C) MR-based association estimates for each CpG site, were considered consistent with associations between ln(creatinine-adjusted urinary As) and CpG sites (from prior EWAS) if their signs were opposite. Study populations were HEALS (n = 379) for DMA% analyses, a subset of the combined cohort with full genotypic data (n = 723) for weighted GP-DMA%, and a combined cohort of HEALS and BEST (n = 772) for MR.

After adjusting for covariates (age, sex, methylation batch, cigarette smoking status, BMI, and education) and log-transformed water arsenic, the directions of DMA% associations were consistent with arsenic exposure associations from prior EWAS (i.e., opposite in sign; see Methods) for 142 out of 221 CpGs (binomial *P* = 1.4 × 10^−5^) (Table 3; Figure 3A). β coefficients for log-transformed DMA had directions consistent with arsenic exposure associations in 146 CpGs (*P* = 1.0 × 10^−6^) (eTable 3; http://links.lww.com/EE/A77, eFigure 2; http://links.lww.com/EE/A77). This pattern confirms the expectation that increasing arsenic exposure and lower DMA% (i.e., higher internal dose of arsenic due to lower metabolism efficiency) should have the same directions of associations with CpGs that are influenced by arsenic exposure (Figure 2).

**Table 3.**
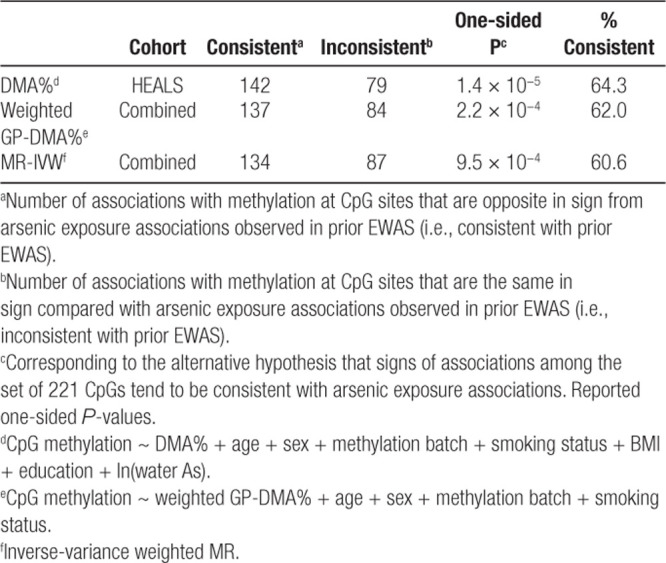
Summary of associations between arsenic metabolism efficiency phenotypes and 221 arsenic-associated CpG sites (compared with results from prior EWAS meta-analysis of urinary arsenic)

### Associations between CpG methylation and genetically predicted DMA%

Using genotype data for 340 individuals from HEALS and 383 from BEST, we analyzed associations between CpG methylation and genetically predicted DMA% (GP-DMA%) in this combined cohort. Stratified results for HEALS and BEST are available in eTable 4; http://links.lww.com/EE/A77. Median weighted GP-DMA% in the HEALS participants was 17.4 (IQR: 16.0, 17.4) and 17.4 in BEST participants (IQR: 14.5, 17.4) (Table 1), relative to an individual carrying zero high-efficiency alleles. After adjusting for sex, age, methylation batch, and cigarette smoking status, weighted GP-DMA% associations with CpGs were consistent with arsenic exposure associations in 137 of the 221 arsenic-associated CpGs (*P* = 0.0002) (Table 3; Figure 3B). Among 41 CpGs that were associated with arsenic exposure based on a Bonferroni *P*-value threshold (*P* < 1.3 × 10^−7^), 29 were associated with weighted GP-DMA% in a consistent direction (*P* = 0.006) (eTable 5; http://links.lww.com/EE/A77). As a sensitivity analysis, we also tested a binary SNP score in 379 individuals from HEALS and 393 from BEST (see Methods). Binary SNP score and CpG associations were consistent with arsenic exposure associations in 148 CpGs (*P* = 2.5 × 10^−7^), and 32 CpGs of Bonferroni *P*-value threshold in EWAS (*P* = 0.0002) (eTable 3; http://links.lww.com/EE/A77, eTable 5; http://links.lww.com/EE/A77, eFigure 3; http://links.lww.com/EE/A77).

### Mendelian randomization

We used a two-sample MR approach based on summary statistics to obtain an MR-based estimate of the association between DMA% and CpG methylation using the 10q24.32 and *FTCD* variants as IVs for DMA%, in the combined HEALS and BEST cohort (n = 772). IVW-MR association estimates for 134 out of 221 CpGs were consistent in direction with arsenic exposure associations (binomial *P* = 0.0010) (Table 3; Figure 3C). When we used the maximum-likelihood MR method as a sensitivity analysis, the effect estimates for 134 CpGs were consistent in direction with arsenic associations (*P* = 0.0010) (eTable 3; http://links.lww.com/EE/A77). Plots for CpGs with the strongest MR effect estimates are included in the Supplement (eFigure 4; http://links.lww.com/EE/A77). When analyses were restricted to 41 CpGs whose associations passed a Bonferroni *P*-value threshold in EWAS, 27 were consistent (*P* = 0.03) (eTable 5; http://links.lww.com/EE/A77). To assess the sensitivity of our MR results to the method of analysis via summary statistics, we also tested associations of CpG methylation levels with individual effect allele counts of 10q24.32 and *FTCD* SNPs (see Methods). Among 221 CpGs, associations between allele scores and CpG methylation were consistent with arsenic exposure associations in 132 CpGs for rs9527 (*P* = 0.0023), 120 for rs11191527 (*P* = 0.11), and 131 for rs61735836 (*P* = 0.0035) (eTable 6; http://links.lww.com/EE/A77, eFigure 5; http://links.lww.com/EE/A77).

### Sensitivity analysis of arsenic-associated CpGs discovered in HEALS

**Figure 4.**
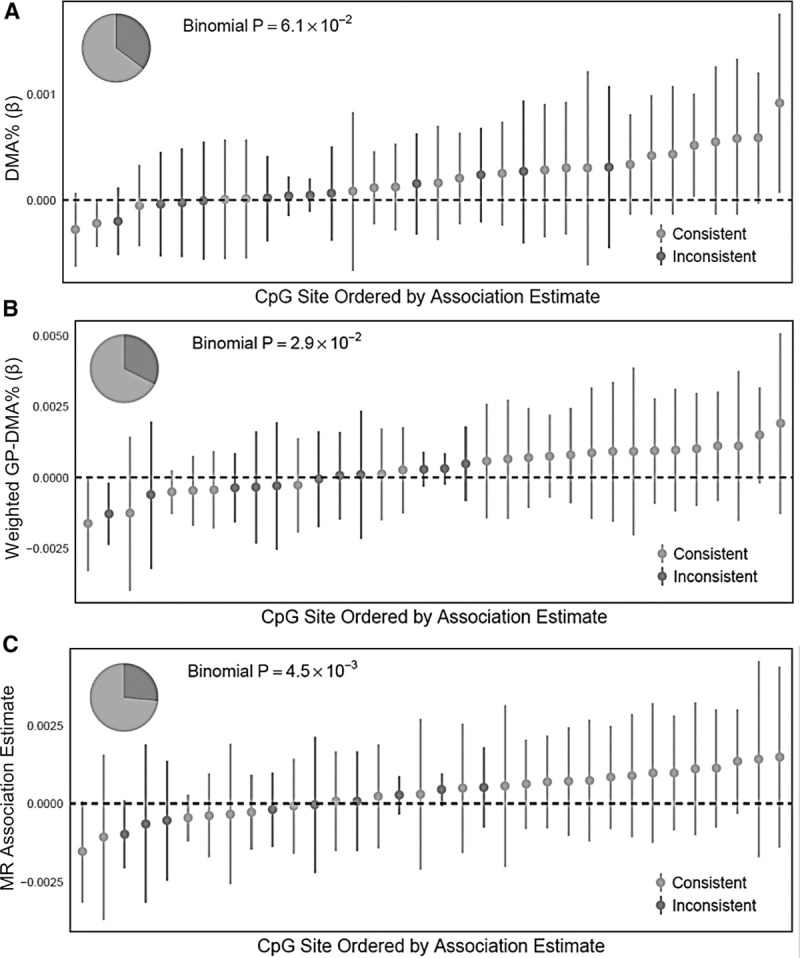
Associations of DMA% and genetically predicted DMA% with DNA methylation at 34 arsenic-associated CpGs discovered in HEALS. (A) DMA%, (B) weighted GP-DMA%, (C) MR. Study populations were HEALS (n = 379) for DMA% and MR analyses, and a subset of HEALS with full genotypic data (n = 340) for weighted GP-DMA%.

We repeated the analyses above, restricting to the set of 34 CpGs measured on the EPIC array and found to be associated (at FDR = 0.05) with urinary arsenic among HEALS participants in the prior EWAS (n = 379 for all analyses except weighted GP-DMA%, where n = 340). Associations with DMA% and log-transformed DMA were consistent with arsenic exposure associations in 22 CpGs (*P* = 0.061) and 24 CpGs (*P* = 0.012), respectively (eTable 7; http://links.lww.com/EE/A77, Figure 4A). Weighted GP-DMA% and binary SNP score associations were consistent with arsenic exposure associations in 23 CpGs (*P* = 0.029) and 27 CpGs (*P* = 0.0004), respectively (eTable 7; http://links.lww.com/EE/A77, Figure 4B). MR estimates were consistent with arsenic exposure associations in 25 CpGs (*P* = 0.0045) (eTable 7; http://links.lww.com/EE/A77, Figure 4C).

## Discussion

In this study, we used an MR approach to provide evidence that supports the hypothesis that arsenic metabolism efficiency is causally related to DNA methylation measured in whole blood at specific CpG sites previously reported to be associated with urinary arsenic. Using a sample of 379 Bangladeshi adults from the HEALS cohort, we demonstrate that the directions of association between DMA% and DNA methylation levels at many arsenic-associated CpG sites tend to be in the opposite direction of the association between urinary arsenic and the same CpGs. Additionally, genetically predicted DMA% (combining information across multiple SNPs) tends to be associated with these CpGs in directions opposite to that of arsenic exposure. Finally, the MR estimates for arsenic-associated CpGs tend to be consistent with the associations between these CpGs and arsenic exposure. These observations support the hypothesis that efficient metabolism of arsenic (i.e., high DMA%) reduces an individual’s internal arsenic dose and should reduce the impact of arsenic on CpGs to which arsenic is causally related.

While the genetics of arsenic metabolism and the associations of arsenic exposure with DNA methylation have been studied previously, in this study, we use the unique approach of applying MR to estimate the association between arsenic metabolism efficiency and methylation at specific CpG sites. The random assignment of study participants’ genotypes minimizes the risk of bias in the association of arsenic metabolism with CpG methylation due to confounding (under the assumption that *AS3MT* and *FTCD* genotypes are associated with CpG methylation only through their effect on arsenic metabolism). This article does not provide strong evidence that arsenic metabolism efficiency affects methylation at any specific CpG site (largely due to lack of statistical power for tests of individual CpGs). These results may not generalize to tissue types other than blood or to other arsenic-exposed populations.

Although the binomial tests of directional consistency are consistent with a causal influence of arsenic metabolism on some fraction of the CpG sites tested, it is unclear if the assumptions of MR are fully satisfied for any individual CpG. MR provides valid evidence supporting causal effects if IVs are (1) associated with the risk factor (DMA%), (2) not associated with confounders of the association between the risk factor and outcome (CpG methylation), and (3) independent of the outcome conditional on the risk factor and confounders.^29^ The third assumption (exclusion restriction) requires that the impact of 10q24.32 and *FTCD* variants on methylation levels of the CpG site be completely mediated by arsenic metabolism efficiency, and this assumption cannot be proven.^29^ Because only three genetic variants are known to be independently associated with DMA%, the number of IVs is insufficient to conduct Egger regression and test for violations of the exclusion restriction. However, the role of *AS3MT* in arsenic metabolism is well-established, providing strong biologic plausibility for a specific effect of these SNPs on arsenic metabolism, even though the SNPs’ biologic mechanisms are not completely understood. The mechanism linking *FTCD* to arsenic metabolism is less clear, but it is potentially related to folate metabolism and the availability of methyl groups for arsenic metabolism.^32^ Given the biologic plausibility of these IVs with respect to arsenic metabolism and the consistency of our results between *AS3MT* SNP rs9527 and *FTCD* SNP rs61735836, we feel that violations of the MR assumptions in this work are unlikely.^31^ Finally, the directions of association for rs11191527 with CpGs were not skewed either positively or negatively. This result may call into question the validity of rs11191527 as an IV. However, the power of our study for detecting associations between rs11191527 and each CpG is limited by the relatively weak association of rs11191527 with DMA%.

An additional limitation of our MR approach is that this approach cannot identify critical periods of this life course in which arsenic exposure affects DNA methylation, nor can we estimate the latency period of such effects. Applying MR only allows us to estimate the impact of lifelong differences in arsenic metabolism efficiency on DNA methylation.

The results of this article build upon those of the EWAS in which these arsenic-associated CpGs were identified, providing evidence for a potential mechanism for arsenic-induced carcinogenesis. Enrichment analyses in the prior EWAS have shown that CpGs annotated to CpG shores, DNase I hypersensitive sites, and enhancers are over-represented among arsenic-associated CpGs identified in the meta-analysis of HEALS and BEST.^21^ These genomic regions have important roles in transcription regulation, and DNA methylation in these regions is more variable and may be more sensitive to environmental exposures.^43^ This results from this article provide additional evidence to supporting studies to further investigate arsenic-associated DNA methylation as a mediator between arsenic exposure and associated diseases and health effects.

This study provides evidence suggesting that a substantial fraction of the associations between arsenic exposure and whole blood DNA methylation observed in prior EWAS represent a causal impact of arsenic, mediated by arsenic metabolism efficiency, on the epigenome. Our MR approach in the context of EWAS is particularly novel, providing an example that can inform future attempts at causal inference in this field and studies of potential molecular effects of environmental exposures. Future studies using larger sample sizes obtained from multiple arsenic-exposed populations should provide more robust MR evidence supporting causal effects of arsenic on specific CpG sites and a more comprehensive understanding of the mechanisms of arsenic-associated disease risk.

## Conflict of interest statement

The authors declare that they have no financial conflict of interest with regard to the content of this report.

## Acknowledgments

We would like to acknowledge the HEALS and BEST study participants and research staff for their contributions to these cohorts and Dr. M. Kibriya for his contributions to generating the DNA methylation data utilized in this analysis.

## Supplementary Material


