# Supplementary material for: Assessing the impact of arsenic metabolism efficiency on DNA methylation using Mendelian randomization
Source: Environ Epidemiol. 2020 Mar 20;4(2):e083. doi: 10.1097/EE9.0000000000000083 (PMC7147391; doi:10.1097/EE9.0000000000000083)
Supplement: Supplementary file 1 [file ee9-4-e083-s001.docx]

Table of Contents

**eTable 1.** CpG sites discovered in HEALS with the EPIC array.

**eTable 2.** CpG sites discovered in meta-analysis of HEALS and BEST.

**eTable 3.** Summary of sensitivity analyses of arsenic-associated CpG sites discovered in meta-analysis.

**eTable 4.** Summary of cohort-stratified analyses of arsenic-associated CpG sites discovered in meta-analysis.

**eTable 5.** Summary of analyses of CpG sites discovered in meta-analysis associated with arsenic based on a Bonferroni P-value threshold.

**eTable 6.** Summary of individual SNP analyses of arsenic-associated CpG sites discovered in meta-analysis.

**eTable 7.** Summary of analyses of arsenic-associated CpG sites discovered in HEALS.

**eFigure 1.** DMA% and ln(DMA) versus arsenic exposure in HEALS participants.

**eFigure 2.** Associations of ln(DMA) with DNA methylation at 221 arsenic-associated CpGs discovered in meta-analysis.

**eFigure 3.** Associations of binary SNP score with DNA methylation at 221 arsenic-associated CpGs discovered in meta-analysis.

**eFigure 4.** Mendelian randomization and forest plots for CpGs with strongest effect estimates.

**eFigure 5.** Associations of each SNP genotype with DNA methylation at 221 arsenic-associated CpGs discovered in meta-analysis.

| **eTable 1.** CpG sites discovered in HEALS with the EPIC array. | | | | | | | |
| --- | --- | --- | --- | --- | --- | --- | --- |
| Name | Chromosome | Position | CpG Location | UCSC Gene Name | UCSC Relation to Gene | Direction of Effect | P |
| cg01912040 | 17 | 1106553 | Shore |  |  | ↓ | 4.2 x 10^-13^ |
| cg05962511 | 10 | 102730022 | Shore |  |  | ↓ | 2.8 x 10^-12^ |
| cg10003262 | 17 | 1106589 | Shore |  |  | ↓ | 2.5 x 10^-11^ |
| cg17420142 | 18 | 32702783 |  | MAPRE2 | Body | ↓ | 3.1 x 10^-10^ |
| cg06466147 | 1 | 155188982 |  | GBAP1 | Body | ↓ | 6.2 x 10^-9^ |
| cg09082427 | 9 | 140349184 | Shore | NSMF | Body | ↓ | 9.9 x 10^-9^ |
| cg04193083 | 17 | 41323562 | Shore | NBR1 | 5'UTR | ↑ | 3.3 x 10^-8^ |
| cg19534475 | 3 | 141632139 |  | ATP1B3 | Body | ↑ | 9.6 x 10^-8^ |
| cg12608784 | 9 | 140349197 | Shore | NSMF | Body | ↓ | 1.2 x 10^-7^ |
| cg14891900 | 17 | 76341204 | Shelf |  |  | ↑ | 1.3 x 10^-7^ |
| cg11308227 | 17 | 79202435 |  | ENTHD2 | 3'UTR | ↓ | 1.3 x 10^-7^ |
| cg13832772 | 4 | 186283800 |  | SNX25 | Body | ↓ | 1.7 x 10^-7^ |
| cg15108641 | 10 | 99263320 | Shelf | UBTD1 | Body | ↑ | 2.5 x 10^-7^ |
| cg09658504 | 9 | 140349188 | Shore | NSMF | Body | ↓ | 3.0 x 10^-7^ |
| cg05438461 | 15 | 40401720 | Shore | BMF | TSS1500 | ↑ | 3.5 x 10^-7^ |
| cg10663081 | 20 | 36837543 |  |  |  | ↓ | 3.7 x 10^-7^ |
| cg08759026 | 11 | 69061454 |  | MYEOV | TSS200 | ↓ | 6.7 x 10^-7^ |
| cg11644394 | 7 | 144148615 |  |  |  | ↑ | 7.5 x 10^-7^ |
| cg05428706 | 10 | 102730130 | Shore |  |  | ↓ | 8.2 x 10^-7^ |
| cg12865207 | 3 | 138669373 | Shore | FOXL2NB | Body | ↑ | 1.0 x 10^-6^ |
| cg09183146 | 16 | 1429863 | Island | UNKL | TSS200 | ↓ | 1.2 x 10^-6^ |
| cg04622454 | 9 | 140349128 | Shore | NELF | Body | ↓ | 1.3 x 10^-6^ |
| cg06381803 | 19 | 46119475 | Island | EML2 | Body | ↓ | 1.4 x 10^-6^ |
| cg02772605 | 1 | 28912323 | Shelf |  |  | ↑ | 1.4 x 10^-6^ |
| cg10283165 | 19 | 17375666 |  | USHBP1 | TSS200 | ↓ | 1.4 x 10^-6^ |
| cg04891961 | 17 | 27939900 | Island | ANKRD13B | Body | ↓ | 1.4 x 10^-6^ |
| cg12746706 | 6 | 169276508 |  |  |  | ↓ | 1.5 x 10^-6^ |
| cg02330195 | 10 | 73342047 |  | CDH23 | Body | ↑ | 1.5 x 10^-6^ |
| cg08077890 | 18 | 157838 | Shore | USP14 | TSS1500 | ↑ | 1.6 x 10^-6^ |
| cg20433952 | 17 | 55607898 |  | MSI2 | Body | ↓ | 1.9 x 10^-6^ |
| cg05646745 | 10 | 135172466 | Shore | FUOM | TSS1500 | ↑ | 1.9 x 10^-6^ |
| cg10185759 | 12 | 60366859 |  |  |  | ↓ | 1.9 x 10^-6^ |
| cg22345623 | 9 | 125050297 |  | MRRF | Body | ↓ | 2.0 x 10^-6^ |
| cg13480898 | 19 | 10195914 | Shore | C19orf66 | TSS1500 | ↓ | 2.0 x 10^-6^ |

| **eTable 2.** CpG sites discovered in meta-analysis. | | | | | | | |
| --- | --- | --- | --- | --- | --- | --- | --- |
| **Name** | **Chromosome** | **Position** | **CpG Location** | **UCSC Gene Name** | **UCSC Relation to Gene** | **Direction of Effect** | **P** |
| cg01912040 | 17 | 1106553 | Shore |  |  | ↓↓ | 3.3 x 10^-17^ |
| cg10003262 | 17 | 1106589 | Shore |  |  | ↓↓ | 1.9 x 10^-15^ |
| cg05962511 | 10 | 102730022 | Shore |  |  | ↓↓ | 2.1 x 10^-13^ |
| cg13480898 | 19 | 10195914 | Shore | C19orf66 | TSS1500 | ↓↓ | 4.1 x 10^-13^ |
| cg07207669 | 1 | 155102388 | Shore | EFNA1 | Body | ↓↓ | 3.0 x 10^-12^ |
| cg01225779 | 5 | 179238472 | Shelf | SQSTM1 | 5'UTR | ↓↓ | 1.0 x 10^-11^ |
| cg06381803 | 19 | 46119475 | Island | EML2 | Body | ↓↓ | 4.8 x 10^-11^ |
| cg09183146 | 16 | 1429863 | Island | UNKL | TSS200 | ↓↓ | 1.4 x 10^-10^ |
| cg08759026 | 11 | 69061454 |  | MYEOV | TSS200 | ↓↓ | 2.8 x 10^-10^ |
| cg17489312 | 1 | 9376039 |  | SPSB1 | 5'UTR | ↓↓ | 2.9 x 10^-10^ |
| cg00472758 | 16 | 2552820 | Shelf | TBC1D24 | 3'UTR | ↓↓ | 5.6 x 10^-10^ |
| cg05428706 | 10 | 102730130 | Shore |  |  | ↓↓ | 5.8 x 10^-10^ |
| cg05425326 | 16 | 58439361 |  | GINS3 | 3'UTR | ↑↑ | 6.1 x 10^-10^ |
| cg26435149 | 3 | 55605611 |  | ERC2 | 3'UTR | ↓↓ | 7.3 x 10^-10^ |
| cg17393635 | 19 | 49843565 | Island | CD37 | Body | ↓↓ | 1.2 x 10^-9^ |
| cg13223043 | 1 | 26492308 | Shore |  |  | ↓↓ | 2.5 x 10^-9^ |
| cg03871754 | 17 | 79320652 | Island |  |  | ↓↓ | 3.3 x 10^-9^ |
| cg19240637 | 2 | 7172297 | Island | RNF144A | Body | ↓↓ | 3.7 x 10^-9^ |
| cg07782285 | 19 | 13085442 |  | DAND5 | 3'UTR | ↓↓ | 7.5 x 10^-9^ |
| cg26390598 | 21 | 41032396 |  | B3GALT5 | 5'UTR | ↑↑ | 1.0 x 10^-8^ |
| cg04622454 | 9 | 140349128 | Shore | NELF | Body | ↓↓ | 1.1 x 10^-8^ |
| cg22959742 | 10 | 13913931 |  | FRMD4A | Body | ↑↑ | 1.3 x 10^-8^ |
| cg00281776 | 2 | 209224225 | Shore |  |  | ↓↓ | 1.8 x 10^-8^ |
| cg12261095 | 19 | 7764345 |  | FCER2 | Body | ↓↓ | 2.0 x 10^-8^ |
| cg14718533 | 10 | 33355576 |  |  |  | ↑↑ | 2.1 x 10^-8^ |
| cg04459545 | 19 | 17375685 |  | USHBP1 | TSS200 | ↓↓ | 3.1 x 10^-8^ |
| cg14145338 | 9 | 139649039 |  | LCN8 | Body | ↓↓ | 3.3 x 10^-8^ |
| cg04920032 | 12 | 50262986 |  | FAIM2 | 3'UTR | ↓↓ | 3.5 x 10^-8^ |
| cg18413900 | 12 | 58160989 | Shore | CYP27B1 | TSS200 | ↑↑ | 3.6 x 10^-8^ |
| cg01757312 | 13 | 112720565 | Island | SOX1 | TSS1500 | ↑↑ | 3.7 x 10^-8^ |
| cg05816193 | 6 | 26018127 | Shelf | HIST1H1A | TSS200 | ↓↓ | 4.4 x 10^-8^ |
| cg07367302 | 1 | 19967428 | Shelf |  |  | ↓↓ | 4.6 x 10^-8^ |
| cg02306995 | 3 | 122635049 | Shelf | SEMA5B | Body | ↓↓ | 5.5 x 10^-8^ |
| cg24318728 | 17 | 39649283 |  |  |  | ↓↓ | 5.9 x 10^-8^ |
| cg04875062 | 1 | 17305562 | Shore | MFAP2 | 5'UTR | ↓↓ | 6.0 x 10^-8^ |
| cg13764516 | 9 | 139648911 |  | LCN8 | 3'UTR | ↓↓ | 6.5 x 10^-8^ |
| cg23050300 | 1 | 3281321 |  | PRDM16 | Body | ↓↓ | 8.4 x 10^-8^ |
| cg18050715 | 13 | 97996992 | Shore | MBNL2 | Body | ↑↑ | 8.7 x 10^-8^ |
| cg04826368 | 6 | 27130208 |  |  |  | ↓↓ | 1.0 x 10^-7^ |
| cg08596618 | 1 | 24275885 |  |  |  | ↓↓ | 1.1 x 10^-7^ |
| cg27092191 | 16 | 31884699 | Shore | ZNF267 | TSS1500 | ↓↓ | 1.2 x 10^-7^ |
| cg06378491 | 11 | 64564012 |  | MAP4K2 | Body | ↓↓ | 1.5 x 10^-7^ |
| cg23233742 | 5 | 139077894 | Shore |  |  | ↓↓ | 1.6 x 10^-7^ |
| cg04543901 | 17 | 63519783 |  |  |  | ↓↓ | 2.1 x 10^-7^ |
| cg11921539 | 11 | 116328665 |  |  |  | ↓↓ | 2.2 x 10^-7^ |
| cg25542319 | 5 | 112540429 |  | MCC | Body | ↑↑ | 2.2 x 10^-7^ |
| cg14983362 | 11 | 118901610 | Island | SLC37A4 | 1stExon,  5'UTR,  TSS1500 | ↑↑ | 2.8 x 10^-7^ |
| cg17442683 | 1 | 8664311 |  | RERE | Body | ↑↑ | 3.5 x 10^-7^ |
| cg06673536 | 19 | 45213941 | Shelf | CEACAM16 | 3'UTR | ↓↓ | 3.7 x 10^-7^ |
| cg10753966 | 3 | 55556197 |  | ERC2 | 3'UTR | ↑↑ | 4.0 x 10^-7^ |
| cg05099952 | 16 | 30367546 | Shore | CD2BP2 | TSS1500 | ↓↓ | 4.2 x 10^-7^ |
| cg07580762 | 1 | 161171810 | Island | NDUFS2 | 5'UTR,  TSS200 | ↑↑ | 4.8 x 10^-7^ |
| cg16423910 | 19 | 49843627 | Island | CD37 | 3'UTR | ↓↓ | 5.5 x 10^-7^ |
| cg06383241 | 12 | 116997022 |  | MAP1LC3B2 | TSS200 | ↓↓ | 5.7 x 10^-7^ |
| cg19270739 | 1 | 1368846 | Shore |  |  | ↓↓ | 5.9 x 10^-7^ |
| cg02314339 | 10 | 91020653 |  |  |  | ↑↑ | 6.0 x 10^-7^ |
| cg14583825 | 19 | 54393040 | Island | PRKCG | Body | ↓↓ | 7.1 x 10^-7^ |
| cg14580628 | 5 | 43602116 | Shore | NNT | TSS1500 | ↓↓ | 7.9 x 10^-7^ |
| cg06186150 | 1 | 2458298 | Shore | PANK4 | TSS1500 | ↑↑ | 8.3 x 10^-7^ |
| cg02158978 | 12 | 133134938 | Shore | FBRSL1 | Body | ↓↓ | 8.4 x 10^-7^ |
| cg24262469 | 3 | 156391694 | Shore | TIPARP /  LOC100287227 | TSS1500 /  Body | ↑↑ | 8.6 x 10^-7^ |
| cg04246167 | 3 | 58984465 |  | C3orf67 | 5'UTR | ↓↓ | 8.7 x 10^-7^ |
| cg20697394 | 12 | 132918305 | Shelf |  |  | ↓↓ | 9.3 x 10^-7^ |
| cg16313343 | 14 | 105768109 | Shore | BRF1 | TSS1500 | ↓↓ | 1.0 x 10^-6^ |
| cg07568588 | 1 | 88338001 |  |  |  | ↓↓ | 1.0 x 10^-6^ |
| cg07240000 | 1 | 17878538 |  | ARHGEF10L | 5'UTR | ↓↓ | 1.1 x 10^-6^ |
| cg20433952 | 17 | 55607898 |  | MSI2 | Body | ↓↓ | 1.2 x 10^-6^ |
| cg14430937 | 12 | 120729982 |  |  |  | ↑↑ | 1.2 x 10^-6^ |
| cg05824218 | 17 | 38499096 | Shore | RARA | 1stExon,  Body | ↓↓ | 1.2 x 10^-6^ |
| cg04605617 | 1 | 20501558 |  | PLA2G2C | 1stExon | ↑↑ | 1.2 x 10^-6^ |
| cg22325145 | 22 | 38825918 | Shore | KCNJ4 | 5'UTR | ↓↓ | 1.2 x 10^-6^ |
| cg03460109 | 4 | 7427313 |  | SORCS2 | Body | ↓↓ | 1.2 x 10^-6^ |
| cg12731773 | 11 | 846055 | Shelf | TSPAN4 | 5'UTR | ↓↓ | 1.2 x 10^-6^ |
| cg25260176 | 12 | 57569940 | Island | LRP1 | Body | ↓↓ | 1.3 x 10^-6^ |
| cg08677954 | 6 | 31604108 |  | BAT2 | Body | ↑↑ | 1.5 x 10^-6^ |
| cg08684991 | 12 | 11662075 |  |  |  | ↓↓ | 1.5 x 10^-6^ |
| cg10959408 | 1 | 54017944 |  | GLIS1 | Body | ↓↓ | 1.6 x 10^-6^ |
| cg10250177 | 1 | 32739752 | Shore | LCK | 5'UTR,  1stExon | ↓↓ | 1.7 x 10^-6^ |
| cg07252792 | 22 | 21987048 | Shelf | CCDC116 | TSS200 | ↓↓ | 1.8 x 10^-6^ |
| cg11152412 | 15 | 74927688 |  | EDC3 | Body | ↑↑ | 1.9 x 10^-6^ |
| cg27554551 | 16 | 4421486 | Shore | VASN /  CORO7 | TSS1500 /  Body | ↓↓ | 1.9 x 10^-6^ |
| cg10817916 | 17 | 61511069 | Shore | CYB561 | 3'UTR | ↓↓ | 2.1 x 10^-6^ |
| cg18394552 | 5 | 159428643 |  |  |  | ↓↓ | 2.2 x 10^-6^ |
| cg01806956 | 1 | 9460830 |  |  |  | ↓↓ | 2.3 x 10^-6^ |
| cg03221025 | 16 | 88955224 | Shore | CBFA2T3 | Body | ↓↓ | 2.4 x 10^-6^ |
| cg22603569 | 19 | 3388047 | Island | NFIC | Body | ↓↓ | 2.5 x 10^-6^ |
| cg00022866 | 11 | 64108440 | Shore | CCDC88B | Body | ↓↓ | 2.5 x 10^-6^ |
| cg10139742 | 16 | 56352151 |  | GNAO1 | Body | ↑↑ | 2.8 x 10^-6^ |
| cg08217545 | 19 | 3388013 | Island | NFIC | Body | ↓↓ | 2.8 x 10^-6^ |
| cg25737313 | 19 | 12899557 | Shore |  |  | ↓↓ | 2.9 x 10^-6^ |
| cg08227353 | 10 | 102821670 | Island | KAZALD1 | 1stExon,  5'UTR | ↓↓ | 3.1 x 10^-6^ |
| cg17934790 | 19 | 14693742 |  | CLEC17A | TSS200 | ↓↓ | 3.1 x 10^-6^ |
| cg06121226 | 4 | 72134060 |  | SLC4A4 | Body | ↓↓ | 3.2 x 10^-6^ |
| cg07262457 | 3 | 128777371 |  |  |  | ↓↓ | 3.3 x 10^-6^ |
| cg19046904 | 22 | 46426201 | Shelf |  |  | ↓↓ | 3.3 x 10^-6^ |
| cg07121900 | 13 | 50706912 | Shore |  |  | ↑↑ | 3.4 x 10^-6^ |
| cg17897187 | 1 | 14923736 | Shore | KIAA1026 | TSS1500 | ↑↑ | 3.4 x 10^-6^ |
| cg08241514 | 22 | 50438769 | Island | IL17REL | Body | ↓↓ | 3.4 x 10^-6^ |
| cg02856716 | 1 | 18993307 |  | PAX7 | Body | ↓↓ | 3.4 x 10^-6^ |
| cg11024682 | 17 | 17730094 | Shelf | SREBF1 | Body | ↓↓ | 3.5 x 10^-6^ |
| cg04418434 | 6 | 7110773 | Shore | RREB1 | 5'UTR | ↑↑ | 3.6 x 10^-6^ |
| cg25881170 | 3 | 107810507 | Island | CD47 | TSS1500 | ↑↑ | 3.6 x 10^-6^ |
| cg21691116 | 1 | 161171819 | Island | NDUFS2 | 5'UTR,  TSS200 | ↑↑ | 3.7 x 10^-6^ |
| cg24251439 | 19 | 1112327 | Island | SBNO2 | Body | ↓↓ | 3.8 x 10^-6^ |
| cg08810397 | 16 | 1521676 | Shelf | CLCN7 | Body | ↓↓ | 4.0 x 10^-6^ |
| cg02159996 | 6 | 89927233 |  | GABRR1 | 5'UTR,  1stExon | ↓↓ | 4.0 x 10^-6^ |
| cg14165660 | 10 | 112889349 |  |  |  | ↓↓ | 4.1 x 10^-6^ |
| cg23006567 | 19 | 53966842 | Shelf |  |  | ↓↓ | 4.1 x 10^-6^ |
| cg14320320 | 3 | 16577787 |  |  |  | ↓↓ | 4.1 x 10^-6^ |
| cg15096140 | 2 | 192109731 | Shore | MYO1B | TSS1500 | ↑↑ | 4.2 x 10^-6^ |
| cg24906420 | 12 | 133133512 | Shore | FBRSL1 | Body | ↓↓ | 4.2 x 10^-6^ |
| cg19753867 | 20 | 35383166 |  | DSN1 | Body | ↑↑ | 4.2 x 10^-6^ |
| cg12232388 | 12 | 125027851 | Shore |  |  | ↓↓ | 4.3 x 10^-6^ |
| cg16049890 | 5 | 139123186 | Shelf |  |  | ↓↓ | 4.4 x 10^-6^ |
| cg22360765 | 2 | 47298563 |  | TTC7A | Body | ↑↑ | 4.6 x 10^-6^ |
| cg20692268 | 1 | 25358981 |  |  |  | ↓↓ | 4.8 x 10^-6^ |
| cg27521562 | 17 | 1107460 | Shore |  |  | ↓↓ | 4.8 x 10^-6^ |
| cg20589883 | 4 | 111115135 | Shelf | ELOVL6 | Body | ↓↓ | 4.9 x 10^-6^ |
| cg15501231 | 6 | 31737630 |  | C6orf27 | Body | ↓↓ | 5.1 x 10^-6^ |
| cg06369349 | 19 | 49503178 |  | RUVBL2 | Body | ↑↑ | 5.4 x 10^-6^ |
| cg24686236 | 5 | 14323412 |  | TRIO | Body | ↓↓ | 5.5 x 10^-6^ |
| cg16688112 | 16 | 88922774 | Island | GALNS /  TRAPPC2L | Body /  TSS1500 | ↓↓ | 5.7 x 10^-6^ |
| cg01695994 | 17 | 80246403 | Shelf |  |  | ↓↓ | 6.0 x 10^-6^ |
| cg05655534 | 2 | 62798448 |  |  |  | ↑↑ | 6.1 x 10^-6^ |
| cg09766547 | 7 | 30465150 |  | NOD1 | 3'UTR | ↓↓ | 6.1 x 10^-6^ |
| cg14176088 | 1 | 9785289 |  | PIK3CD | Body | ↑↑ | 6.3 x 10^-6^ |
| cg18642369 | 13 | 99651231 |  | DOCK9 | Body | ↑↑ | 6.4 x 10^-6^ |
| cg03031932 | 16 | 81547138 |  | CMIP | Body | ↓↓ | 6.6 x 10^-6^ |
| cg17176619 | 13 | 99218657 |  | STK24 | Body | ↑↑ | 6.6 x 10^-6^ |
| cg01008405 | 5 | 139039026 | Shore | CXXC5 | 5'UTR | ↓↓ | 6.7 x 10^-6^ |
| cg07286682 | 15 | 69447132 |  | MIR548H4 | Body | ↓↓ | 7.2 x 10^-6^ |
| cg02873991 | 12 | 25151263 |  | C12orf77 | TSS1500 | ↓↓ | 7.3 x 10^-6^ |
| cg10816760 | 6 | 142890973 |  | LOC153910 | Body | ↓↓ | 7.5 x 10^-6^ |
| cg26906737 | 1 | 212541551 |  | TMEM206 | Body | ↑↑ | 7.6 x 10^-6^ |
| cg20793420 | 22 | 38861635 | Shelf |  |  | ↓↓ | 7.6 x 10^-6^ |
| cg12746706 | 6 | 169276508 |  |  |  | ↓↓ | 7.6 x 10^-6^ |
| cg16743273 | 19 | 2076833 | Island | MOBKL2A | Body | ↓↓ | 7.6 x 10^-6^ |
| cg04193083 | 17 | 41323562 | Shore | NBR1 | 5'UTR | ↑↑ | 7.7 x 10^-6^ |
| cg11992015 | 2 | 110876674 | Shelf |  |  | ↑↑ | 7.9 x 10^-6^ |
| cg08530064 | 7 | 150598393 |  |  |  | ↓↓ | 7.9 x 10^-6^ |
| cg23889047 | 1 | 7705756 |  | CAMTA1 | Body | ↓↓ | 8.3 x 10^-6^ |
| cg11831006 | 3 | 125100635 |  |  |  | ↓↓ | 8.4 x 10^-6^ |
| cg24539599 | 5 | 112824920 | Shore | MCC | TSS1500 | ↑↑ | 8.9 x 10^-6^ |
| cg09742751 | 20 | 16553921 | Island | KIF16B | 1stExon,  5'UTR | ↓↓ | 9.0 x 10^-6^ |
| cg09449232 | 12 | 106135284 |  |  |  | ↑↑ | 9.2 x 10^-6^ |
| cg00591660 | 16 | 2478353 | Shore | CCNF | TSS1500 | ↓↓ | 9.4 x 10^-6^ |
| cg08949143 | 13 | 30947713 |  | LOC100188949 | Body | ↓↓ | 9.5 x 10^-6^ |
| cg02402423 | 19 | 41255444 | Shore | C19orf54 /  SNRPA | 1stExon /  TSS1500 | ↓↓ | 9.7 x 10^-6^ |
| cg26354367 | 6 | 30010457 |  | NCRNA00171 | Body | ↓↓ | 9.7 x 10^-6^ |
| cg13271643 | 13 | 21036583 |  | CRYL1 | Body | ↓↓ | 9.9 x 10^-6^ |
| cg16417374 | 1 | 26098310 | Shore | MAN1C1 | Body | ↓↓ | 1.0 x 10^-5^ |
| cg22563815 | 15 | 78856949 | Shore | CHRNA5 | TSS1500 | ↓↓ | 1.1 x 10^-5^ |
| cg17861836 | 17 | 79202505 |  | C17orf56 | 3'UTR | ↓↓ | 1.1 x 10^-5^ |
| cg23907108 | 11 | 64405993 | Island | NRXN2 | Body | ↑↑ | 1.1 x 10^-5^ |
| cg05018460 | 15 | 80688078 |  |  |  | ↑↑ | 1.1 x 10^-5^ |
| cg16668176 | 19 | 5308893 |  | PTPRS | 5'UTR | ↓↓ | 1.1 x 10^-5^ |
| cg20429981 | 1 | 1609972 | Shore | CDK11B /  LOC728661 | Body,  5'UTR /  5'UTR | ↓↓ | 1.1 x 10^-5^ |
| cg01994902 | 16 | 1576069 | Shore | IFT140 | Body | ↓↓ | 1.2 x 10^-5^ |
| cg07870074 | 10 | 706006 |  | DIP2C /  C10orf108 | Body /  Body | ↓↓ | 1.2 x 10^-5^ |
| cg00129811 | 6 | 116726398 |  | DSE | Body | ↓↓ | 1.3 x 10^-5^ |
| cg15226659 | 19 | 4552594 | Island | SEMA6B | Body | ↓↓ | 1.3 x 10^-5^ |
| cg22617002 | 13 | 112275633 | Island |  |  | ↓↓ | 1.4 x 10^-5^ |
| cg22137772 | 3 | 150755800 |  | CLRN1OS | Body | ↓↓ | 1.4 x 10^-5^ |
| cg05048624 | 1 | 20441579 |  | PLA2G2D | Body | ↓↓ | 1.4 x 10^-5^ |
| cg08417595 | 19 | 3811002 | Island | ZFR2 | Body | ↓↓ | 1.4 x 10^-5^ |
| cg13800769 | 12 | 4916913 | Shore | KCNA6 | TSS1500 | ↓↓ | 1.4 x 10^-5^ |
| cg04514392 | 3 | 107086058 |  |  |  | ↓↓ | 1.4 x 10^-5^ |
| cg20421058 | 4 | 7337225 |  | SORCS2 | Body | ↓↓ | 1.4 x 10^-5^ |
| cg22001211 | 2 | 61766532 | Shore | XPO1 | TSS1500 | ↓↓ | 1.5 x 10^-5^ |
| cg18753841 | 11 | 8680469 |  | TRIM66 | TSS200 | ↑↑ | 1.5 x 10^-5^ |
| cg21646084 | 2 | 16802843 |  | FAM49A | 5'UTR | ↓↓ | 1.5 x 10^-5^ |
| cg08817422 | 6 | 30654638 | Island | KIAA1949 | 5'UTR,  1stExon | ↓↓ | 1.5 x 10^-5^ |
| cg21896766 | 8 | 114444395 | Shore | CSMD3 | Body | ↑↑ | 1.6 x 10^-5^ |
| cg11793449 | 17 | 76313872 | Shelf |  |  | ↓↓ | 1.6 x 10^-5^ |
| cg01616876 | 12 | 113544928 | Shelf | RASAL1 | Body | ↓↓ | 1.6 x 10^-5^ |
| cg16163382 | 2 | 37938640 |  |  |  | ↓↓ | 1.6 x 10^-5^ |
| cg17375396 | 11 | 67202808 | Shore | RPS6KB2 | 3'UTR | ↓↓ | 1.6 x 10^-5^ |
| cg19377250 | 7 | 100463206 | Shore | SLC12A9 | Body | ↓↓ | 1.6 x 10^-5^ |
| cg07528595 | 12 | 54814557 | Shore |  |  | ↓↓ | 1.7 x 10^-5^ |
| cg24712249 | 1 | 155830294 | Island | SYT11 | Body | ↓↓ | 1.7 x 10^-5^ |
| cg06827038 | 17 | 48912952 |  | WFIKKN2 | 1stExon,  5'UTR | ↓↓ | 1.7 x 10^-5^ |
| cg01154283 | 2 | 36603543 |  | CRIM1 | Body | ↓↓ | 1.7 x 10^-5^ |
| cg13148151 | 3 | 45637978 | Shelf | LIMD1 | Body | ↓↓ | 1.7 x 10^-5^ |
| cg26829395 | 15 | 40217855 |  |  |  | ↓↓ | 1.7 x 10^-5^ |
| cg17743381 | 1 | 39024825 |  |  |  | ↓↓ | 1.8 x 10^-5^ |
| cg08977611 | 20 | 42953248 | Shelf |  |  | ↓↓ | 1.8 x 10^-5^ |
| cg00013899 | 1 | 64992433 |  | CACHD1 | Body | ↓↓ | 1.9 x 10^-5^ |
| cg13729816 | 19 | 52954722 | Island |  |  | ↓↓ | 1.9 x 10^-5^ |
| cg21900616 | 22 | 20286845 | Island |  |  | ↓↓ | 1.9 x 10^-5^ |
| cg00722097 | 6 | 111195976 | Island | AMD1 | TSS200 | ↓↓ | 2.0 x 10^-5^ |
| cg24277586 | 3 | 52099561 |  | C3orf74 | TSS1500 | ↓↓ | 2.0 x 10^-5^ |
| cg05483509 | 16 | 2563435 | Island | ATP6V0C | TSS1500 | ↓↓ | 2.0 x 10^-5^ |
| cg07636952 | 10 | 124596397 |  | CUZD1 | Body | ↑↑ | 2.1 x 10^-5^ |
| cg08986840 | 8 | 38409865 |  |  |  | ↓↓ | 2.1 x 10^-5^ |
| cg04202267 | 2 | 169431900 |  | LASS6 | Body | ↓↓ | 2.1 x 10-^5^ |
| cg18132851 | 6 | 152085641 |  | ESR1 | 5'UTR | ↑↑ | 2.1 x 10^-5^ |
| cg19507267 | 3 | 108896930 |  | C3orf66 | TSS200 | ↓↓ | 2.1 x 10^-5^ |
| cg14588606 | 17 | 39183560 |  | KRTAP1-5 | TSS200 | ↑↑ | 2.2 x 10^-5^ |
| cg20729813 | 4 | 7502429 |  | SORCS2 | Body | ↑↑ | 2.3 x 10^-5^ |
| cg15033653 | 12 | 113587581 | Shelf | CCDC42B | TSS200 | ↓↓ | 2.3 x 10^-5^ |
| cg05339515 | 16 | 68624615 |  |  |  | ↑↑ | 2.3 x 10^-5^ |
| cg10529613 | 14 | 25287305 |  | STXBP6 | Body | ↓↓ | 2.4 x 10^-5^ |
| cg25921609 | 17 | 8379225 | Shore | MYH10 | Body | ↓↓ | 2.4 x 10^-5^ |
| cg14172849 | 14 | 104171259 |  | XRCC3 | Body | ↓↓ | 2.4 x 10^-5^ |
| cg12464638 | 11 | 844400 | Shore | TSPAN4 | TSS200,  5'UTR | ↓↓ | 2.4 x 10^-5^ |
| cg27064708 | 14 | 69030612 |  | RAD51L1 | Body | ↓↓ | 2.5 x 10^-5^ |
| cg04929932 | 1 | 91195683 | Shelf |  |  | ↑↑ | 2.5 x 10^-5^ |
| cg21807240 | 14 | 23425770 | Shore | HAUS4 | 5'UTR | ↓↓ | 2.6 x 10^-5^ |
| cg04331601 | 19 | 17633880 | Shore | FAM129C | TSS1500 | ↓↓ | 2.6 x 10^-5^ |
| cg17744604 | 1 | 206946166 |  | IL10 | TSS1500 | ↓↓ | 2.6 x 10^-5^ |
| cg01881062 | 1 | 6660403 | Shore | KLHL21 | Body | ↓↓ | 2.6 x 10^-5^ |
| cg22356061 | 1 | 227954102 |  | SNAP47 | Body | ↓↓ | 2.6 x 10^-5^ |
| cg16808156 | 20 | 3835875 |  | MAVS | Body | ↑↑ | 2.6 x 10^-5^ |
| cg13543355 | 14 | 77350013 |  |  |  | ↓↓ | 2.6 x 10^-5^ |
| cg20629315 | 2 | 43685377 |  | THADA | Body | ↓↓ | 2.7 x 10^-5^ |
| cg27016544 | 17 | 10002002 |  | GAS7 | Body | ↓↓ | 2.7 x 10^-5^ |
| cg13357922 | 10 | 135251512 | Shelf |  |  | ↑↑ | 2.7 x 10^-5^ |
| cg14211332 | 1 | 117255389 |  |  |  | ↑↑ | 2.7 x 10^-5^ |
| cg27572855 | 1 | 25598939 |  | RHD | TSS200 | ↓↓ | 2.8 x 10^-5^ |
| cg05708512 | 5 | 31731391 |  |  |  | ↓↓ | 2.8 x 10^-5^ |
| cg25598890 | 12 | 117501447 |  | TESC | Body | ↓↓ | 2.8 x 10^-5^ |
|  | | | | | | | |

| **eTable 3.** Summary of sensitivity analyses of arsenic-associated CpG sites discovered in meta-analysis. | | | | | | |
| --- | --- | --- | --- | --- | --- | --- |
|  | **Cohort** | **Consistent^a^** | **Inconsistent^b^** | **P^c^** | **% Consistent** |  |
| ln(DMA)^d^ | HEALS | 146 | 75 | 1.0 x 10^-6^ | 66.1% |  |
| MR Max-Likelihood^e^ | Combined | 134 | 87 | 9.5 x 10^-4^ | 60.6% |  |
| Binary SNP Score^f,g^ | Combined | 148 | 73 | 2.5 x 10^-7^ | 67.0% |  |
| ^a^Number of associations with methylations of CpG sites that are opposite in sign from arsenic exposure associations.  ^b^Same sign as arsenic exposure associations.  ^c^Relative to the null hypothesis that signs of associations among the set of 221 CpGs do not tend to be consistent with arsenic exposure associations. Reported one-sided p-values.  ^d^CpG methylation ~ ln(DMA) + age + sex + methylation batch + smoking status + BMI + education + ln(water As) + ln(urine As) + ln(urine creatinine).  ^e^Maximum likelihood Mendelian randomization  ^f^CpG methylation ~ binary SNP score + age + sex + methylation batch + smoking status  ^g^Coded as a binary variable indicating 2 copies of the effect allele for both rs9527 and rs61735836. | | | | | | |

| **eTable 4.** Summary of cohort-stratified analyses of arsenic-associated CpG sites discovered in meta-analysis. | | | | | | |
| --- | --- | --- | --- | --- | --- | --- |
|  | **Cohort** | **Consistent^a^** | **Inconsistent^b^** | **P^c^** | **% Consistent** |  |
| Weighted GP-DMA%^d^ | HEALS | 130 | 91 | 5.2 x 10^-3^ | 58.8% |  |
|  | BEST | 134 | 87 | 9.5 x 10^-4^ | 60.6% |  |
| Binary SNP Score^e,f^ | HEALS | 146 | 75 | 1.0 x 10^-6^ | 66.1% |  |
|  | BEST | 108 | 113 | 6.6 x 10^-1^ | 48.9% |  |
| MR-IVW^g^ | HEALS | 131 | 90 | 3.5 x 10^-3^ | 59.3% |  |
|  | BEST | 134 | 87 | 1.0 x 10^-3^ | 60.6% |  |

^a^Number of associations with methylations of CpG sites that are opposite in sign from arsenic exposure associations.

^b^Same sign as arsenic exposure associations.

^c^Relative to the null hypothesis that signs of associations among the set of 221 CpGs do not tend to be consistent with arsenic exposure associations. Reported one-sided p-values.

^d^CpG methylation ~ weighted GP-DMA% + age + sex + methylation batch + smoking status

eCpG methylation ~ binary SNP score + age + sex + methylation batch + smoking status

^f^Coded as a binary variable indicating 2 copies of the effect allele for both rs9527 and rs61735836.

^g^Inverse-variance weighted Mendelian randomization

| **eTable 5.** Summary of analyses of CpG sites discovered in meta-analysis associated with arsenic based on a Bonferroni P-value threshold. | | | | | | |
| --- | --- | --- | --- | --- | --- | --- |
|  | **Cohort** | **Consistent^a^** | **Inconsistent^b^** | **P^c^** | **% Consistent** |  |
| Weighted GP-DMA%^d^ | Combined | 29 | 12 | 5.8 x 10^-3^ | 70.7% |  |
| Binary SNP Score^e,f^ | Combined | 32 | 9 | 2.2 x 10^-4^ | 78.0% |  |
| MR-IVW^g^ | Combined | 27 | 14 | 3.0 x 10^-2^ | 65.9% |  |

^a^Number of associations with methylations of CpG sites that are opposite in sign from arsenic exposure associations.

^b^Same sign as arsenic exposure associations.

^c^Relative to the null hypothesis that signs of associations among the set of 41 CpGs do not tend to be consistent with arsenic exposure associations. Reported one-sided p-values.

^d^CpG methylation ~ weighted GP-DMA% + age + sex + methylation batch + smoking status

eCpG methylation ~ binary SNP score + age + sex + methylation batch + smoking status

^f^Coded as a binary variable indicating 2 copies of the effect allele for both rs9527 and rs61735836.

^g^Inverse-variance weighted Mendelian randomization

| **eTable 6.** Summary of individual SNP analyses of arsenic-associated CpG sites discovered in meta-analysis. | | | | | | |
| --- | --- | --- | --- | --- | --- | --- |
|  | **Cohort** | **Consistent^a^** | **Inconsistent^b^** | **P^c^** | **% Consistent** |  |
| rs9527^d,f^ | HEALS | 108 | 113 | 6.6 x 10^-1^ | 48.9% |  |
|  | Combined | 132 | 89 | 2.3 x 10^-3^ | 59.7% |  |
| rs11191527^e,f^ | HEALS | 94 | 127 | 9.9 x 10^-1^ | 42.5% |  |
|  | Combined | 120 | 101 | 1.1 x 10^-1^ | 54.3% |  |
| rs61735836^d,g^ | HEALS | 136 | 85 | 3.7 x 10^-4^ | 61.5% |  |
|  | Combined | 131 | 90 | 3.5 x 10^-3^ | 59.3% |  |
| ^a^Number of associations with methylations of CpG sites that are opposite in sign from arsenic exposure associations.  ^b^Same sign as arsenic exposure associations.  ^c^Relative to the null hypothesis that signs of associations among the set of 41 CpGs do not tend to be consistent with arsenic exposure associations. Reported one-sided p-values.  ^d^Coded as a binary variable indicating 2 copies of effect allele.  ^e^Coded as a binary variable indicating 1 or 2 copies of effect allele.  ^f^CpG methylation ~ rs9527 + rs11191527 + age + sex + methylation batch + smoking status  ^g^CpG methylation ~ rs61735836 + age + sex + methylation batch + smoking status | | | | | | |

| **eTable 7.** Summary of analyses of arsenic-associated CpG sites discovered in HEALS. | | | | | | |
| --- | --- | --- | --- | --- | --- | --- |
|  | **Cohort** | **Consistent^a^** | **Inconsistent^b^** | **P^c^** | **% Consistent** |  |
| DMA%^d^ | HEALS | 22 | 12 | 6.1 x 10^-2^ | 64.7% |  |
| ln(DMA)^e^ | HEALS | 24 | 10 | 1.2 x 10^-2^ | 70.6% |  |
| Weighted GP-DMA%^f^ | HEALS | 23 | 11 | 2.9 x 10^-2^ | 67.6% |  |
| Binary SNP Score^g,h^ | HEALS | 27 | 7 | 4.0 x 10^-4^ | 79.0% |  |
| MR-IVW^i^ | HEALS | 25 | 9 | 4.5 x 10^-3^ | 73.5% |  |

^a^Number of associations with methylations of CpG sites that are opposite in sign from arsenic exposure associations.

^b^Same sign as arsenic exposure associations.

^c^Relative to the null hypothesis that signs of associations among the set of 34 CpGs do not tend to be consistent with arsenic exposure associations. Reported one-sided p-values.

^d^CpG methylation ~ DMA% + age + sex + methylation batch + smoking status + BMI + education + ln(water As)

^e^CpG methylation ~ ln(DMA) + age + sex + methylation batch + smoking status + BMI + education + ln(water As) + ln(urine As) + ln(urine creatinine).

^f^CpG methylation ~ weighted GP-DMA% + age + sex + methylation batch + smoking status

gCpG methylation ~ binary SNP score + age + sex + methylation batch + smoking status

^h^Coded as a binary variable indicating 2 copies of the effect allele for both rs9527 and rs61735836.

^i^Inverse-variance weighted Mendelian randomization

| 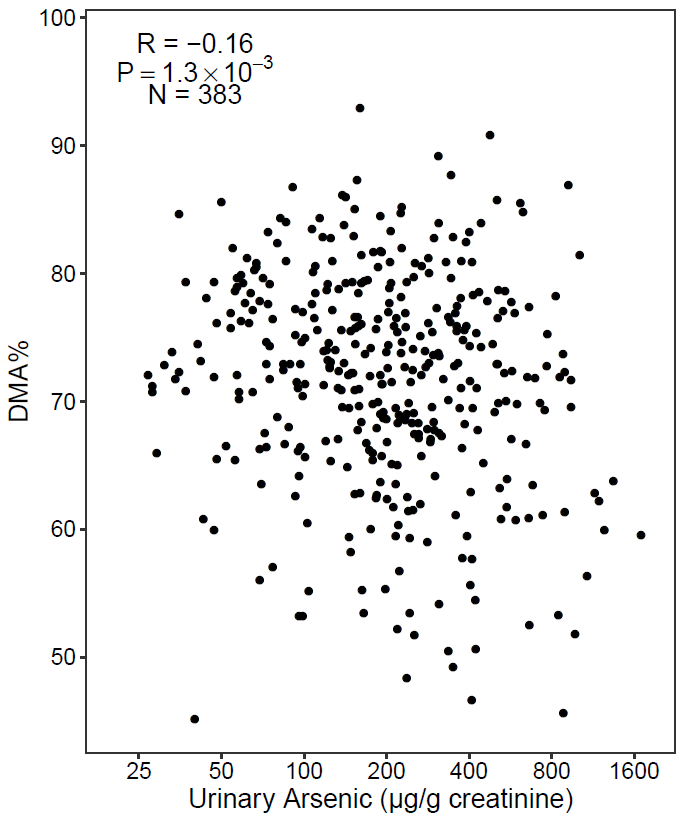 | 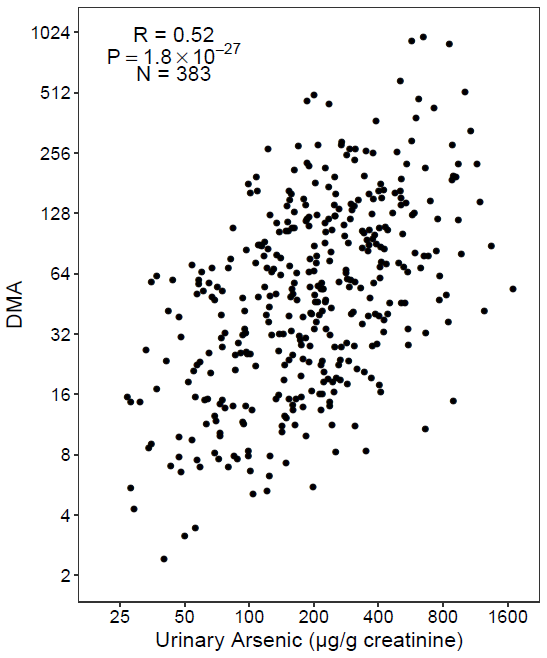 |
| --- | --- |
| **eFigure 1. DMA% and ln(DMA) versus arsenic exposure in HEALS participants (n=383).** Pearson correlations and corresponding p-values were reported. | |

|  |
| --- |
| 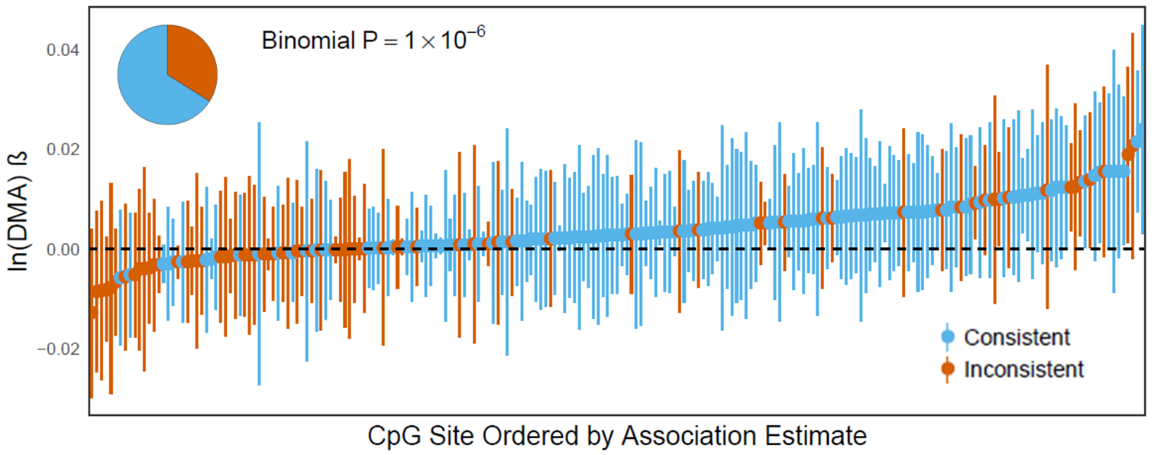**eFigure 2. Associations of ln(DMA) with DNA methylation at 221 arsenic-associated CpGs discovered in meta-analysis.** The study population was HEALS (*n* = 379). |

|  |
| --- |
| 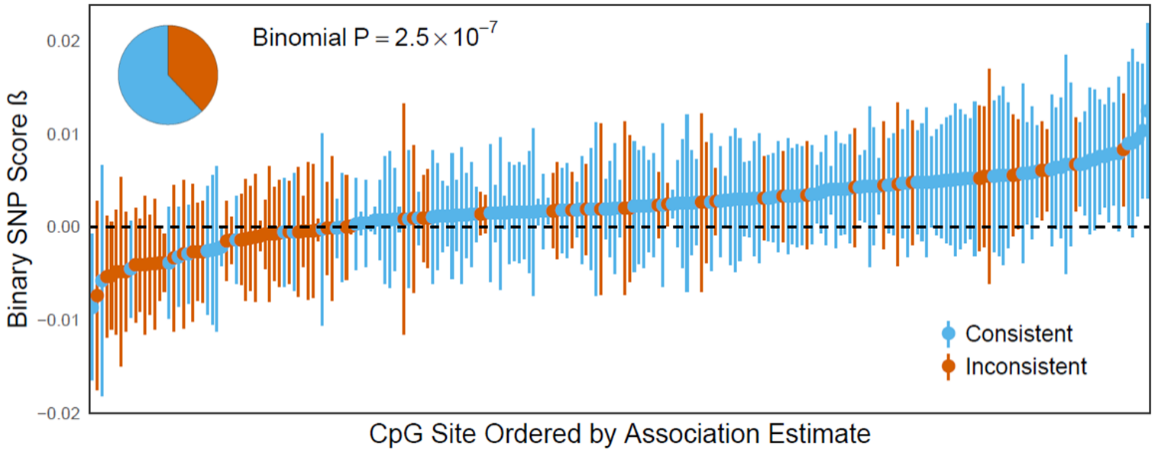**eFigure 3. Associations of binary SNP score with DNA methylation at 221 arsenic-associated CpGs discovered in meta-analysis.** The study population was the combined HEALS and BEST cohort (*n* = 772). |

| 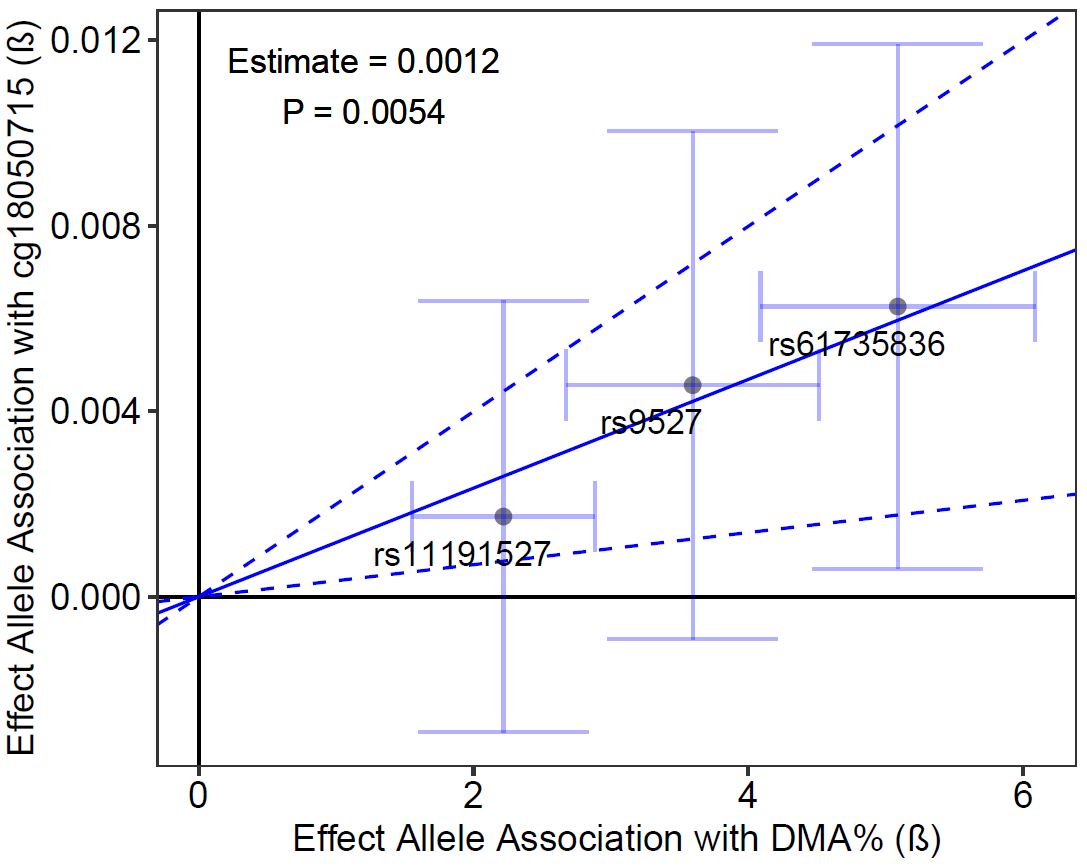A | 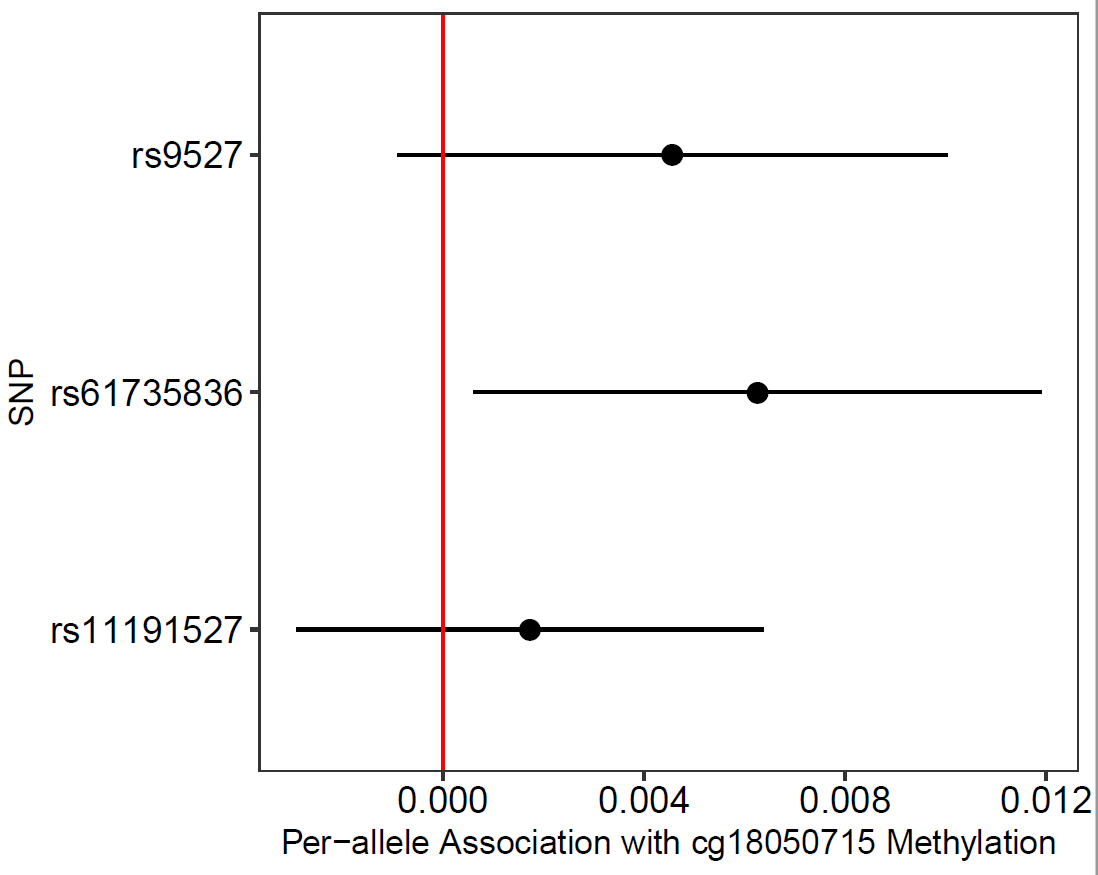 B |
| --- | --- |
| 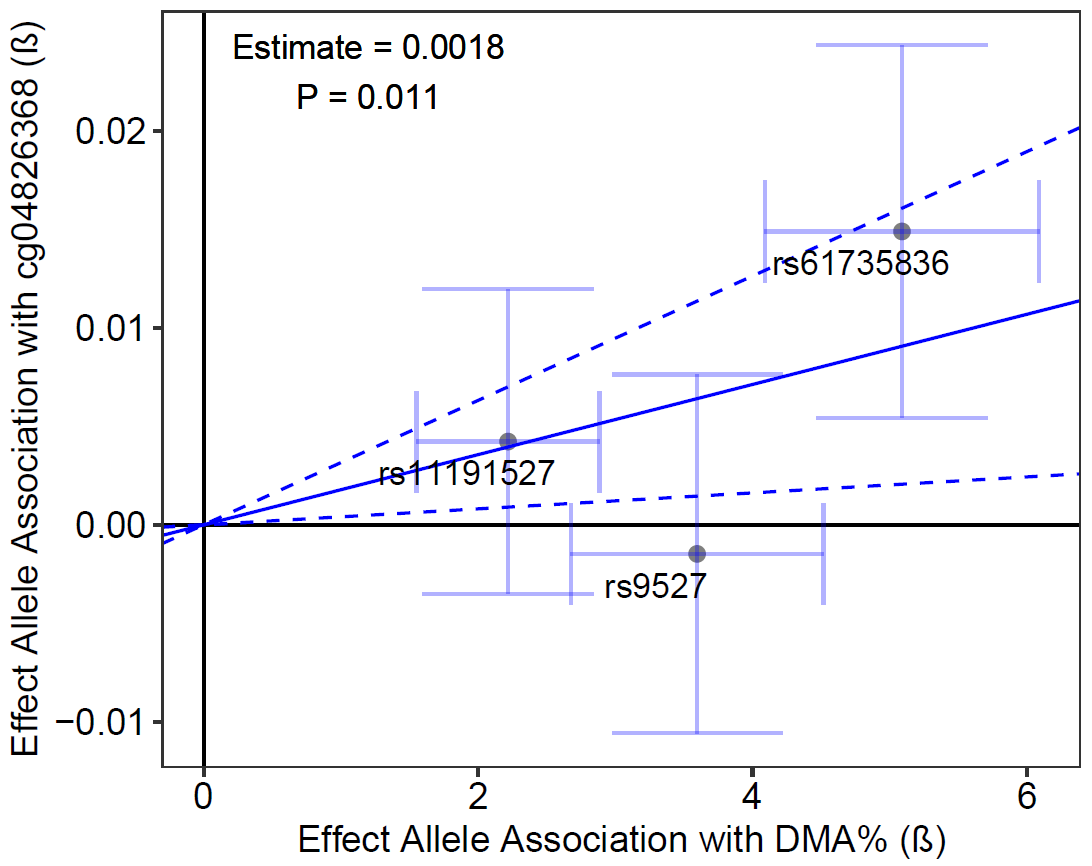  C | 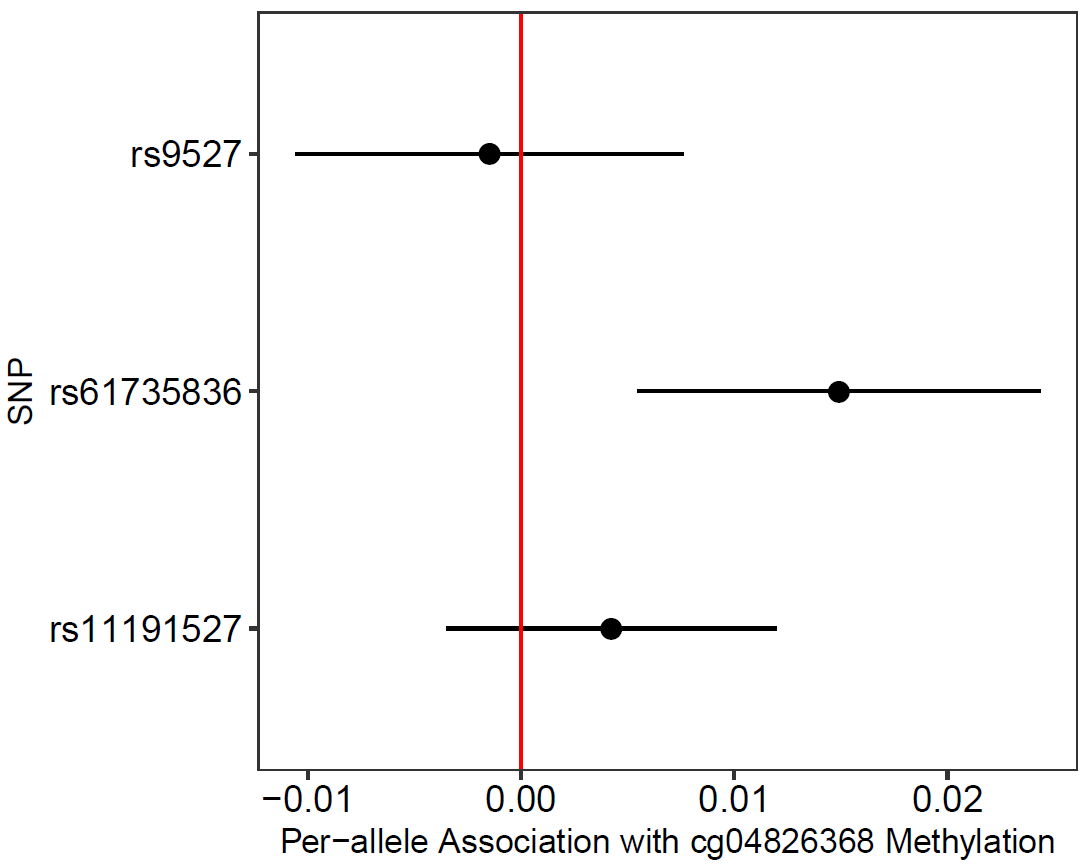  D |
| 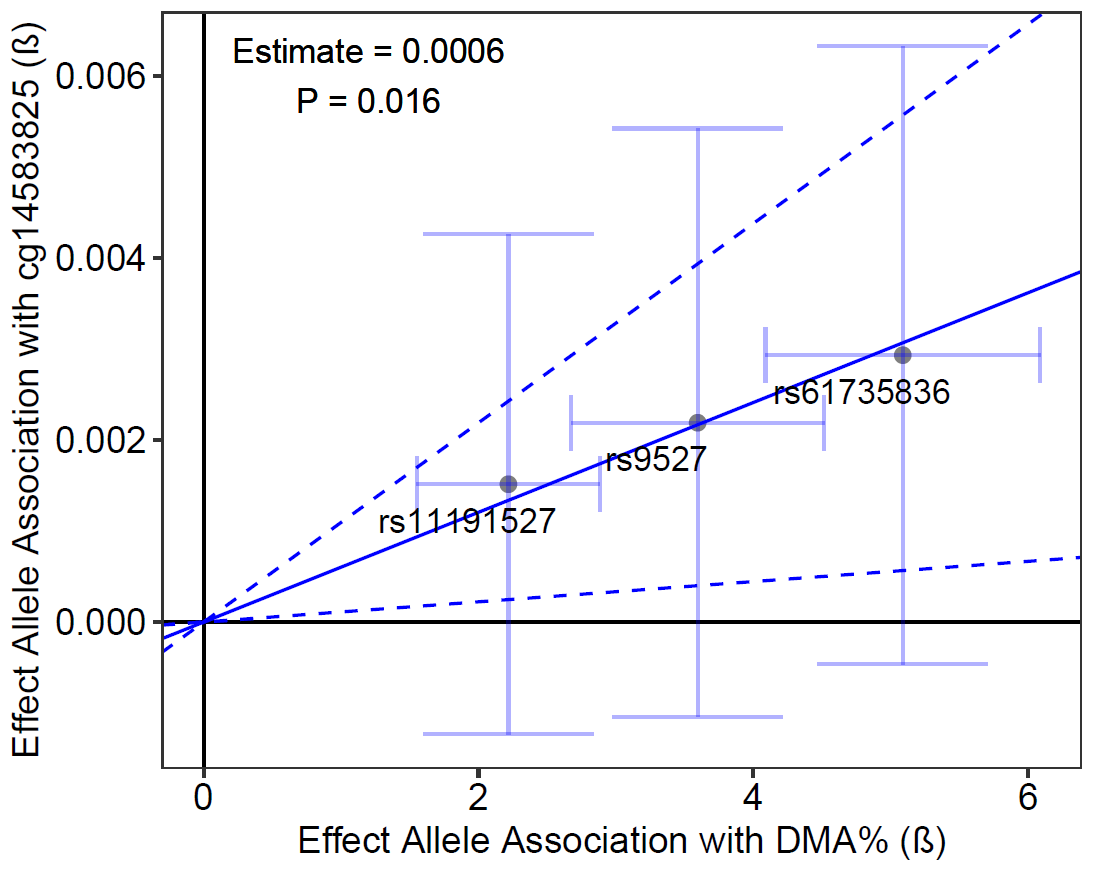  E | 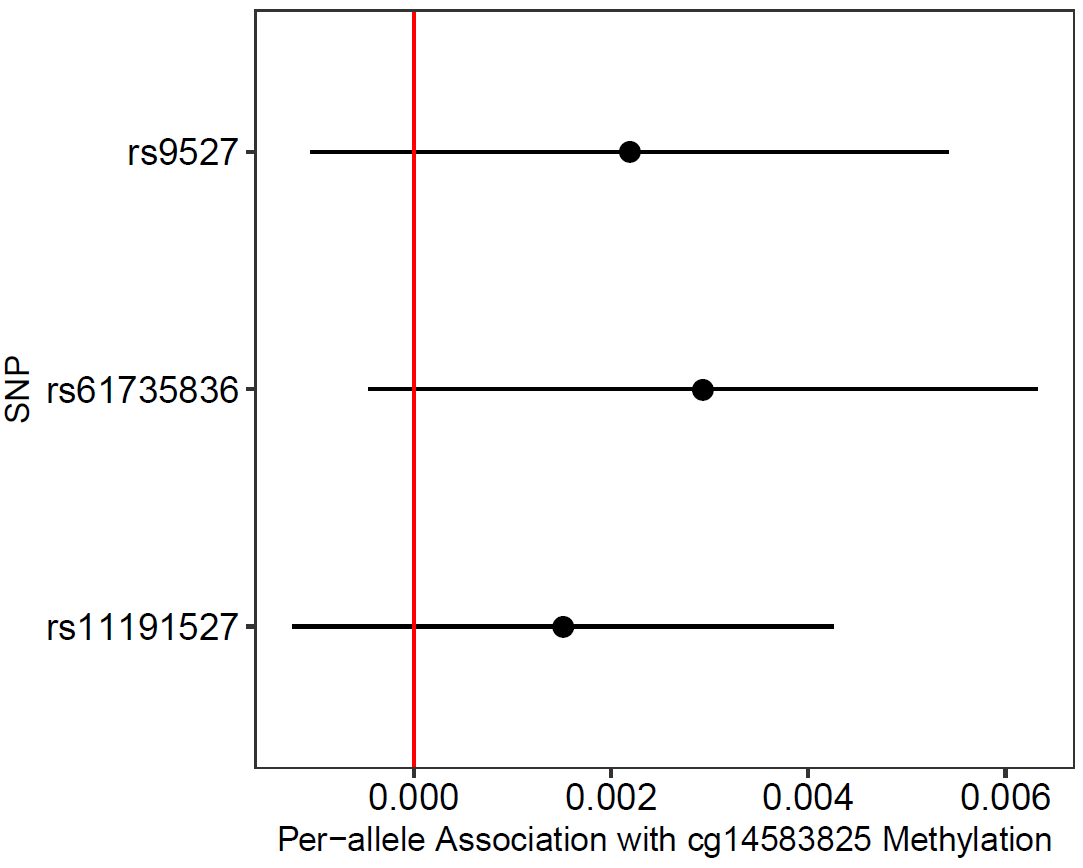  F |
|  |  |
| **eFigure 4. Mendelian randomization and forest plots for CpGs with strongest effect estimates**. (A-B) cg18050715, (C-D) cg04826368, (E-F) cg14583825. (A, C, E) Horizontal and vertical error bars represent the 95% CI for the β coefficient of each SNP’s association with DMA% and CpG methylation, respectively. (B, D, F) Horizontal lines represent the 95% CI for β coefficient of each SNP’s association with CpG methylation. | |

| 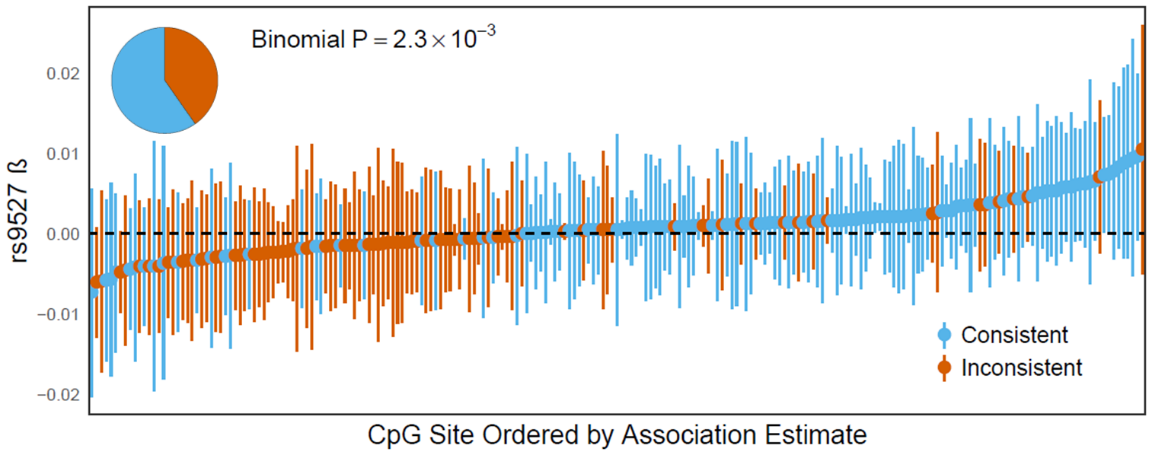A |
| --- |
| 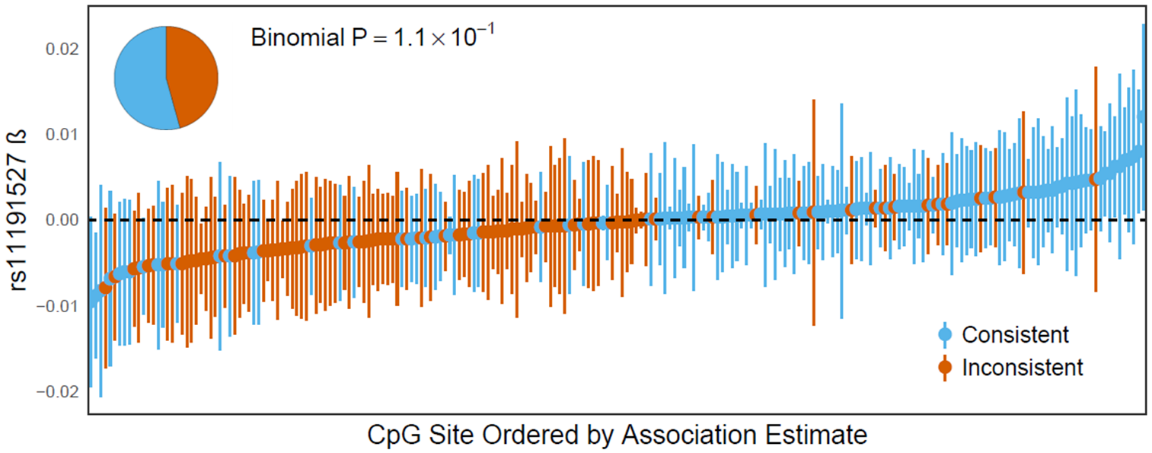B |
| 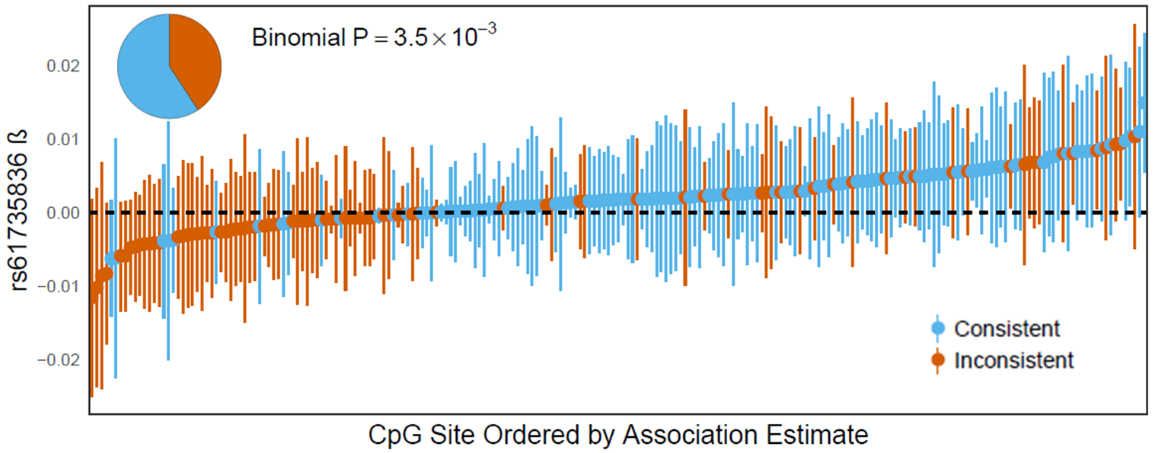C |
| **eFigure 5. Associations of each SNP genotype with DNA methylation at 221 arsenic-associated CpGs discovered in meta-analysis.** (A) rs9527, (B) rs11191527, and (C) rs61735836. Study populations were a combined cohort of HEALS and BEST (*n* = 772) for rs9527 and rs11191527, and a subset of the combined cohort with full genotypic data (*n* = 723) for rs61735836. |
